# Combined Nav1.7–Nav1.8 channel abrogation: Supralinear suppression of excitability in a subpopulation of peripheral sensory neurons

**DOI:** 10.1085/jgp.202614101

**Published:** 2026-10-30

**Authors:** Dmytro Vasylyev, Sidharth Tyagi, Mohammad-Reza Ghovanloo, Peng Zhao, Stephen G. Waxman

**Affiliations:** 1 Department of Neurology, Yale School of Medicine, New Haven, CT, USA; 2 Center for Neuroscience and Regeneration Research, West Haven, CT, USA; 3 Center for Restoration of Nervous System Function, VA Connecticut Healthcare System, West Haven, CT, USA; 4 Department of Anesthesiology, Yale School of Medicine, New Haven, CT, USA

## Abstract

There is a pressing need for nonaddictive pain therapeutics. Despite the genetic validation of peripheral voltage-gated sodium (Nav) channels as targets for non-opioid pain therapy, isoform-selective inhibition of Nav1.7 and Nav1.8 has yielded limited efficacy in clinical studies, with Nav1.8 blockade providing statistically significant but only partial pain relief. This raises the question: if block of Nav1.8 relieves pain only partially, what is needed for better pain relief? In this study, we utilized dynamic clamp, which permits precisely calibrated functional addition or subtraction of the current from any given channel, to systematically interrogate the combinatorial landscape of multiplex abrogation of currents from Nav1.7 and Nav1.8, both major contributors to electrogenesis within dorsal root ganglion neurons. We demonstrate that partial, simultaneous attenuation of both conductances drives a supralinear increase in current threshold in many sensory neurons. Responses to Nav1.8 functional subtraction span a broad spectrum, whereas responses to Nav1.7 subtraction are comparatively uniform. Neurons with greater sensitivity to functional loss of Nav1.8 show greater suppression of excitability during combined Nav1.7–Nav1.8 attenuation, while weak Nav1.8 responders remain comparatively resistant. These findings identify electrogenic dependence on Nav1.8 as a major source of functional heterogeneity in sensory neurons and demonstrate that Nav1.7 acts cooperatively with Nav1.8 to shape the consequences of channel attenuation. Our results indicate that combined Nav1.7–Nav1.8 targeting should outperform isoform-selective block in suppressing firing in a subpopulation of pain-signaling neurons, while not producing universal suppression.

## Teaser

Vasylyev, Tyagi et al. show that Nav1.7 and Nav1.8 sodium channels work cooperatively to control sensory-neuron excitability. Combined partial suppression raises the firing threshold more than expected from either channel alone, but responses vary across neurons, revealing both the potential and limits of dual-channel targeting for pain treatment.

## Introduction

There is a pressing need for nonaddictive pain therapeutics. Within primary sensory neurons, peripheral voltage-gated sodium (Nav) channels Nav1.7 and Nav1.8 have emerged as especially important determinants of nociceptor excitability and have been studied extensively as potential therapeutic targets (Rush et al., 2007; Bennett et al., 2019; Vasylyev et al., 2024; Ghovanloo and Waxman, 2026). Nav1.7 amplifies small subthreshold depolarizations and helps set the gain for action potential (AP) initiation (Cummins et al., 1998), while slowly inactivating Nav1.8, by virtue of its relatively depolarized voltage dependence and rapid recovery from inactivation, supports the regenerative AP upstroke and repetitive firing of pain-signaling neurons (Kocsis and Waxman, 1987; Akopian et al., 1996; Renganathan et al., 2001). The roles of Nav1.7 and Nav1.8 have been substantially genetically validated in a series of pain-related disorders (Dib-Hajj et al., 2005). Whereas gain- and loss-of-function variants of Nav1.7 generally produce pain and pain insensitivity, respectively, both gain- and loss-of-function variants of Nav1.8 have been associated with painful neuropathy, suggesting a more complex genotype–phenotype relationship (Cox et al., 2006; Faber et al., 2012; Kaluza et al., 2018). Still, despite mechanistic and genetic target validation, clinical success with isoform-selective inhibition has been incomplete. Selective Nav1.8 inhibition with VX-548 (suzetrigine) reduces acute postoperative pain in humans, but the magnitude of analgesia was partial (Jones et al., 2023). This raises the question: If blocking of Nav1.8 relieves pain only partially, what is needed for better pain relief?

Recent work in human dorsal root ganglion (DRG) neurons showed that even strong pharmacological inhibition of Nav1.8 reduces, but does not abolish, repetitive firing, in part because excitability can still be supported by other sodium currents, especially Nav1.7 (Stewart et al., 2025; Vasylyev et al., 2025; Waxman and Vasylyev, 2025). These observations suggest that the limited efficacy of monotherapy with Nav blockers may reflect a biophysical constraint imposed by the electrogenic architecture of nociceptors. Prior dynamic clamp and modeling studies have shown that Nav1.7 and Nav1.8 play distinct but intertwined roles during AP electrogenesis, with Nav1.7 contributing strongly near resting potential and Nav1.8 amplifying excitability over a broader depolarized range, particularly during repetitive firing (Han et al., 2015; Vasylyev et al., 2024; Vasylyev et al., 2025). In recent studies, functional subtraction of Nav1.8 conductance has shown that there is a population of weak-responder DRG neurons in which even major reductions in Nav1.8 currents do not produce significant suppression of excitability, suggesting an intrinsic limit to the efficacy of Nav1.8-directed monotherapy (Vasylyev et al., 2025).

In this study, we investigated the effects of combined inhibition of Nav1.7 and Nav1.8, using precisely calibrated dynamic clamp functional abrogation (subtraction) of the Nav1.7 and Nav1.8 currents to systematically examine the effects of combined abrogation of Nav1.7 and Nav1.8 conductances in small DRG neurons from male rats. Dynamic clamp is uniquely suited to this approach because it enables precise, real-time manipulation of specific conductances in intact neurons (Sharp et al., 1993b; Prinz et al., 2004a). This approach allowed us to examine the effect of multiple combinations of paired reduction of Nav1.7 and Nav1.8, studied in the same cell, on excitability of DRG neurons.

Here, we show that partial simultaneous attenuation of Nav1.7 and Nav1.8 produces a supralinear increase in current threshold in a subset of sensory neurons. Importantly, responses to Nav1.8 functional subtraction span a broad spectrum, whereas responses to Nav1.7 subtraction are comparatively uniform across the sampled population. The magnitude of Nav1.7–Nav1.8 synergy likewise varies across neurons, reflecting variability in their underlying electrogenic architecture. Our findings support the idea that combined Nav1.7–Nav1.8 channel targeting can be more effective than isoform-selective inhibition of Nav1.7 or Nav1.8 alone but also suggest that additional conductances must be modulated to fully suppress excitability in the overall population of peripheral pain-signaling neurons.

## Materials and methods

### DRG neuron preparation

Time-pregnant dams were purchased from Inotiv/Envigo and arrived in-house at E17–E18. Pups were maintained with the respective dam, who had free access to food and water. DRGs from male postnatal day 4–5 (P4–P5) Sprague–Dawley rats were harvested and dissociated as reported previously (Dib-Hajj et al., 2009). Briefly, DRGs were harvested and placed in ice-cold complete saline solution (CSS) (in mM: 137 NaCl, 5.3 KCl, 1 MgCl_2_, 25 sorbitol, 3 CaCl_2_, and 10 HEPES, adjusted to pH 7.2 with NaOH). DRGs were then incubated at 37°C for 20-min in CSS containing 1.5 mg/ml Collagenase A (Roche) and 0.6 mM EDTA, followed by 20-min incubation at 37°C in CSS containing 1.5 mg/ml Collagenase D (Roche), 0.6 mM EDTA, and 30 U/ml papain (Worthington Biochemical); the DRGs were then triturated in DRG culture media, supplemented with 1.5 mg/ml low-endotoxin BSA (Sigma-Aldrich) and 1.5 mg/ml trypsin inhibitor (Sigma-Aldrich). DRG culture media was DMEM/F12 (Invitrogen) supplemented with 10% fetal bovine serum (HyClone), 100 U/ml penicillin, 0.1 mg/ml streptomycin (Invitrogen), and 2 mM L-glutamine (Invitrogen). After trituration, 100 μl of cell suspension was seeded directly onto each poly-D-lysine/laminin-coated coverslip (Corning, Discovery Labware) and incubated at 37°C in a 95% air/5% CO2 (vol/vol) incubator. After allowing 45 min for neurons to attach to the coverslips, DRG media supplemented with 50 ng/ml NGF and 50 ng/ml GDNF was added to each well to a final volume of 1.0 ml, and the neurons were maintained at 37°C in a 95% air/5% CO_2_ (vol/vol) incubator until used for electrophysiological experiments. Half of the media supplemented with 50 ng/ml NGF and 50 ng/ml GDNF was changed every other day. Electrophysiological recordings were performed between 3 and 6 days in vitro.

### Electrophysiology and dynamic clamp

**Figure S1.**
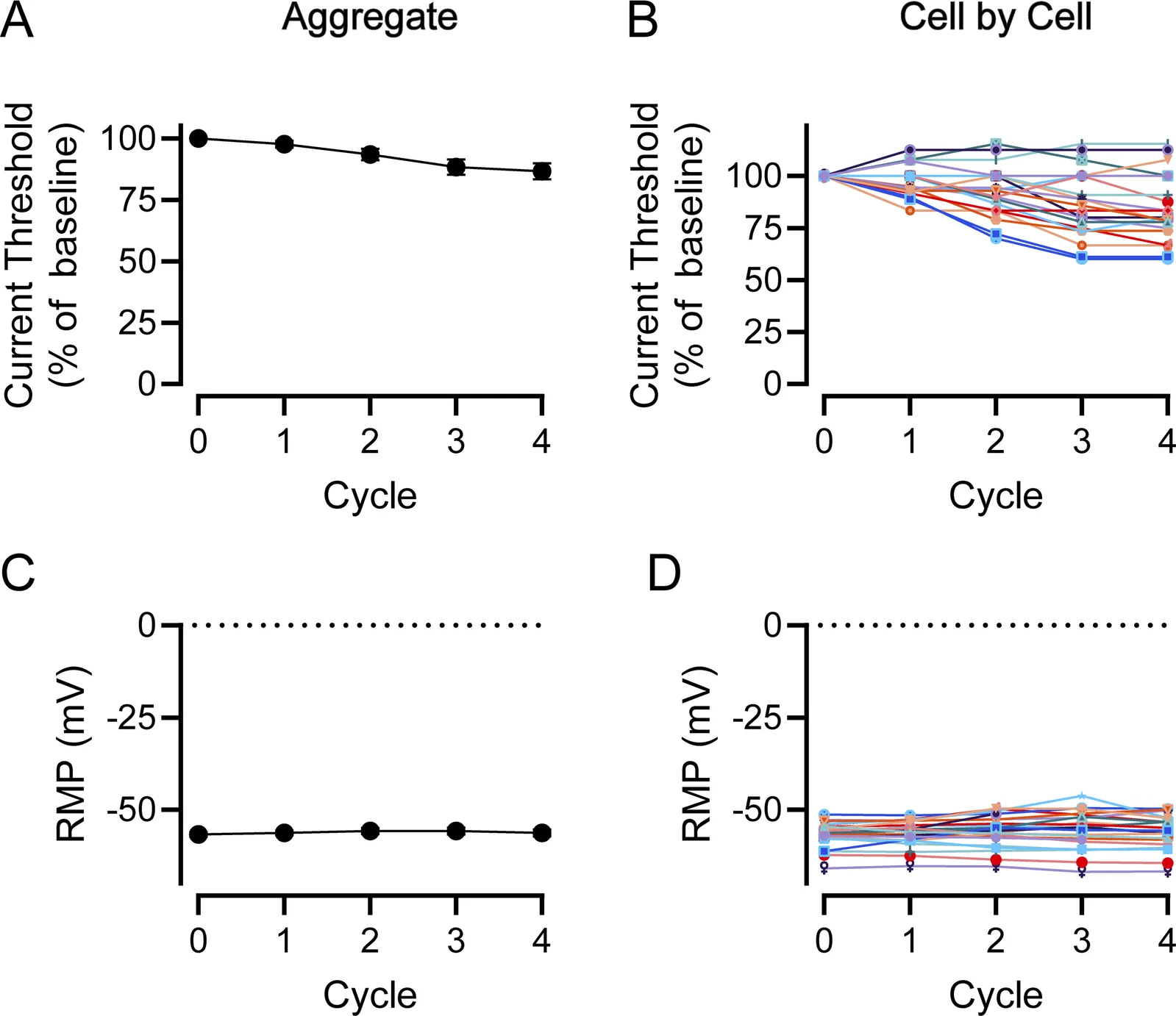
**Stability of electrophysiological parameters during the dynamic-clamp recording protocol.** Control recordings with 0% Nav1.7 and 0% Nav1.8 subtraction were obtained at the beginning of the protocol (cycle 0) and after each block of four matrix recordings (cycles 1–4), spanning the ∼50-min experiment. **(A)** Aggregate current threshold at each control cycle, normalized to the initial control recording. **(B)** Current-threshold trajectories for individual neurons. **(C)** Aggregate RMP across control cycles. **(D)** RMP trajectories for individual neurons. Lines connect repeated measurements from the same neuron, and colors in B and D distinguish individual neurons. Data in A and C are mean ± SEM; *n* = 24 neurons. Stability of the recordings was assessed at the end of each matrix column (every four recordings) by repeating the measurements under control conditions. Thus, a total of 20 recordings were obtained: 16 Matrix recordings and 4 additional control recordings, with the initial control serving as the reference for the first 4 Matrix recordings. If a small drift in measured parameters was observed, it was accounted for, as parameter changes within each matrix column were calculated relative to the most recent control recording.

Small-diameter DRG neurons (soma diameter 24–28 μm, 25.88 ± 1.1 µm, mean ± SD, *n* = 24) were studied in whole-cell configuration under dynamic clamp (Sharp et al., 1993b; Sharp et al., 1993a; Kemenes et al., 2011; Samu et al., 2012; Vasylyev et al., 2014; Takkala and Prescott, 2018; Vasylyev et al., 2023; Vasylyev et al., 2024). Membrane voltage and current recordings were acquired using a HEKA EPC10 USB amplifier and PatchMaster software interfaced with a Cybercyte CIM/V10 dynamic clamp system with 20–25 μs latency. Voltage and current traces were filtered at 3 kHz and digitized at 50 kHz. Recordings were performed at room temperature (21–23°C). Patch pipettes had resistances of 1.5–3 MΩ when filled with intracellular solution containing (in mM): 150 KCl, 0.5 EGTA, 5 HEPES, 3 Mg-ATP, and 5.6 glucose, adjusted to pH 7.3 with KOH, 286–288 mOsm. We used KCl solutions because Cs based solutions would suppress native potassium conductances and thereby fundamentally alter the APs and current threshold measurements under investigation. Extracellular solution was HBSS (Invitrogen, catalog number 14025) (in mM): 1.3 CaCl_2_, 0.5 MgCl_2_, 0.4 MgSO_4_, 5.3 KCl, 0.4 KH_2_PO_4_, 4.2 NaHCO_3_, 138 NaCl, 0.3 Na_2_HPO_4_, 5.6 glucose, and 284–286 mOsm. During current-clamp recordings, neurons were maintained at their native resting membrane potential (RMP) without steady-state current injection. Stability of the recordings (current threshold, RMP, and overshoot) was assessed at the end of each matrix column (every four recordings) by repeating the measurements under control conditions. Thus, a total of 20 recordings were obtained: 16 Matrix recordings and four additional control recordings, with the initial control serving as the reference for the first 4 Matrix recordings. If a small drift in measured parameters was observed, it was accounted for, as parameter changes within each matrix column were calculated relative to the most recent control recording. Cells that did not meet predefined electrophysiological quality criteria (unstable RMP or current threshold, overshoot less than +34 mV, in control) were excluded from analysis. These stability values are presented in aggregate and cell by cell in Fig. S1. RMP was considered unstable if it exhibited a sustained change of >10 mV from the initial control recording. Among the included neurons, the observed maximal RMP deviations (in mV) for all 24 cells were as follows: 1, 2.8, 3.2, 5.5, 4.4, 4.3, 6.5, 3.4, 3.1, 1.1, 2.8, 7.7, 0.8, 1.9, 2.2, 2.7, 1.9, 3, 1.6, 4.2, 1.5, 3.3, 3.4, and 3.1; these values yielded a mean maximal ΔRMP of 3.14 ± 0.34 mV (mean ± SE, *n* = 24). Serial RMP measurements are presented in Fig. S1.

**Figure 1.**
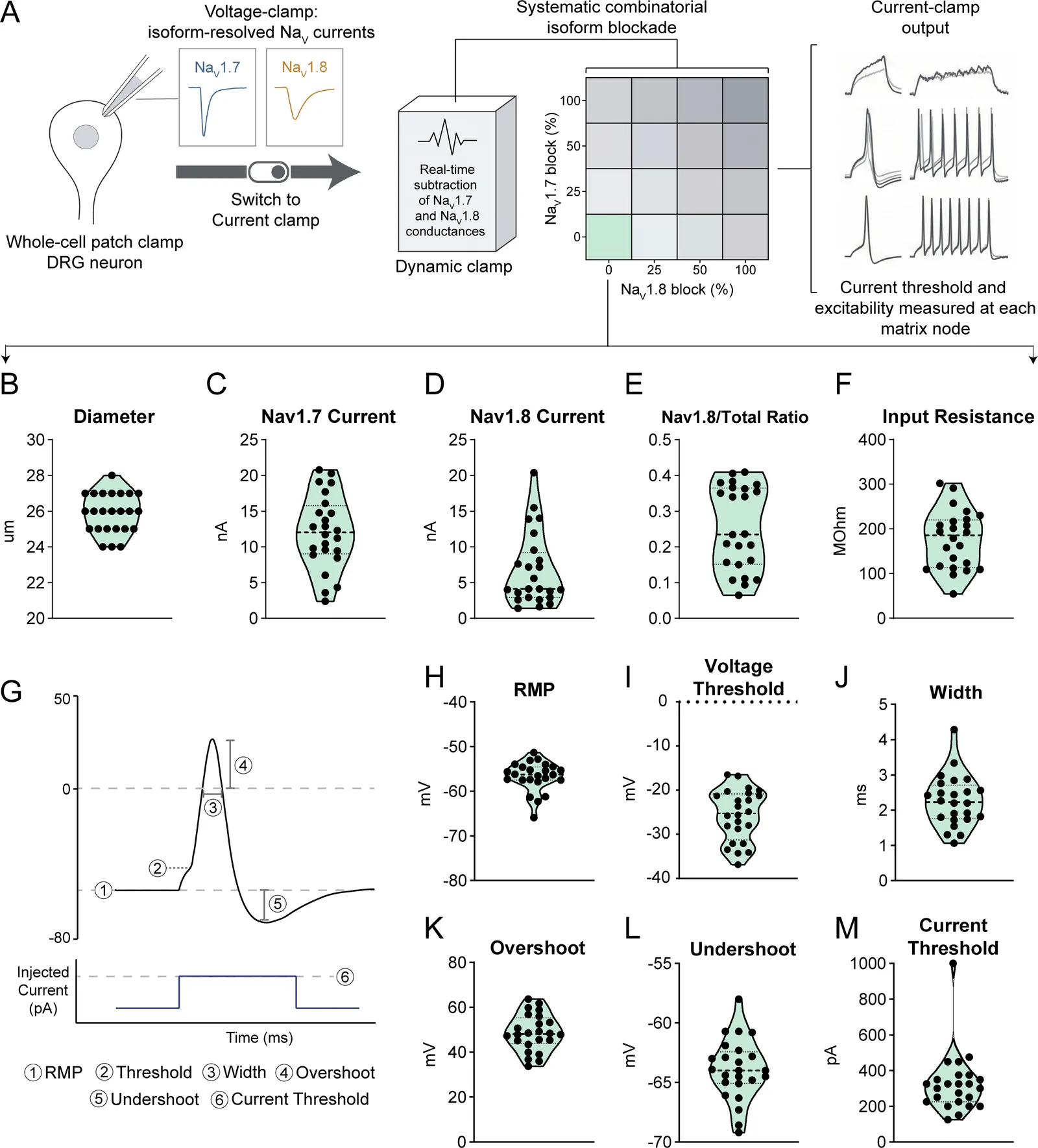
**Population-wide characterization of baseline biophysical parameters. (A)** Overview of the transitions from voltage-clamp characterization to the 16-node current-clamp matrix. **(B)** Distribution of cell sizes within the sampled population, showing a focus on small-to-medium diameter neurons. **(C–E)** Baseline distribution for Nav1.7 and Nav1.8 currents as well as the Nav1.8/total current ratio. **(F)** Input resistance distribution. **(G)** AP parameters measured included RMP, threshold, width, overshoot, undershoot, and current threshold. **(H–M)** Population-wide distributions for RMP (H), threshold (I), AP width (measured at 0 mV) (J), overshoot (K), undershoot (L), and absolute current threshold (M). These metrics establish the baseline state of the cells prior to simulated isoform functional subtraction.

Current threshold was measured using 10-ms depolarizing current injections (thus, “current threshold” here refers to the minimal current required to evoke an AP under this 10-ms stimulation protocol, rather than classical rheobase), and voltage threshold was defined as the point at which the first derivative of the membrane potential began to rise sharply from baseline. Baseline electrophysiological properties (Fig. 1), including RMP, voltage threshold, AP width, overshoot, undershoot, input resistance, and firing pattern, were also quantified. AP width was measured at 0 mV and was considered undefined when the AP peak did not exceed 0 mV. Current threshold and AP peak voltage remained measurable in these recordings. When no AP could be evoked within the applied stimulus range, current threshold was assigned the maximum stimulus strength; these values therefore represent conservative lower-bound estimates.

For each neuron, whole-cell voltage clamp was first used to estimate endogenous Nav1.7 and Nav1.8 current amplitudes. To isolate the Nav1.8 component (hereafter denoted TTX-R*) of the endogenous net sodium current, we applied test voltage pulses from a holding potential of −45 mV, at which other Nav channels were fully inactivated at steady state, while Nav1.8 inactivation remained minimal (Cummins and Waxman, 1997; Sheets et al., 2007; Vasylyev et al., 2024). The TTX-S* component of the sodium current was obtained by point-by-point digital subtraction of the TTX-R* sodium current from the total sodium current evoked at the respective test voltage from a holding potential of −80 mV.

Nav1.7 current was estimated from the tetrodotoxin-sensitive sodium current component based on prior evidence that Nav1.7 constitutes ∼70% of tetrodotoxin-sensitive current in small DRG neurons (Vasylyev et al., 2014); the remaining TTX-sensitive current was not assigned to a specific isoform (which may include Nav1.3 or Nav1.6) or included in the dynamic-clamp subtraction. These experimentally measured sodium current amplitudes were then used to scale Hodgkin–Huxley-based Nav1.7 and Nav1.8 models within the dynamic clamp system by adjusting the maximal conductance (Gmax) for each channel model so that modeled currents matched the magnitude of the endogenous sodium currents recorded from that cell. Dynamic clamp current injected through the recording pipette therefore functionally mimicked the amplitude and timing of native sodium currents while preserving endogenous channel expression and activity.

The kinetic models utilized in this study were derived from the Hodgkin–Huxley framework (Hodgkin and Huxley, 1952), following the equation dx/dt = α_x_(1 − x) − β_x_x, where *x* represents the channel gating variable, and α and β are the forward and backward rate constants (ms^−1^). Sodium current was expressed as I_Na_ = G_max_*m^3^*h*s*(V_m_ − E_Na_), where G_max_ is the maximum conductance, V_m_ is membrane voltage, and E_Na_ (sodium reversal potential) was set at 65 mV. The activation variable is denoted as *m*, while *h* and *s* represent inactivation variables (*s* = 1 for Nav1.8 channels).

To simulate sodium currents, we applied in silico AP clamp protocols (Blair and Bean, 2002; Vasylyev et al., 2014), computing responses offline at 20 μs resolution to match the PatchMaster digitization protocol. Calculations were performed using APSIM, a custom program scripted in LabTalk within the Origin framework (Vasylyev et al., 2014). Hodgkin–Huxley variables, their products (m^3^hs), and corresponding ionic currents were generated computationally using APSIM. In silico modeling of Nav1.7 and Nav1.8 currents was conducted by matching the model’s maximal conductance values to the respective native currents recorded from each cell. The model’s conductance was adjusted to ensure that the peak amplitude of simulated currents aligned with experimentally recorded values under identical voltage protocols. Values of Gmax for Nav1.7 and Nav1.8 are provided in the text as 100% conductance.

The Nav1.7 channel model was from Vasylyev et al. (2014)

$$\alpha_{m}=10.22-\frac{10.22}{1+\exp\left(\frac{V_{m}+7.19}{15.43}\right)},$$

$$\beta_{m}=\frac{23.76}{1+\exp\left(\frac{V_{m}+70.37}{14.53}\right)},$$

$$\alpha_{h}=\frac{0.0744}{1+\exp\left(\frac{V_{m}+99.76}{11.07}\right)},$$

$$\beta_{h}=2.54-\frac{2.54}{1+\exp\left(\frac{V_{m}+7.8}{10.68}\right)},$$

$$\alpha_{s}=0.00003+\frac{0.00092}{1+\exp\left(\frac{V_{m}+93.9}{16.6}\right)},$$

$$\beta_{s}=132.05-\frac{132.05}{1+\exp\left(\frac{V_{m}-384.9}{28.5}\right)}.$$

The Nav1.8 channel model was from Sheets et al. (2007)

$$\alpha_{m}=2.85-\frac{2.839}{1+\exp\left(\frac{V_{m}-1.159}{13.95}\right)},$$

$$\beta_{m}=\frac{7.6205}{1+\exp\left(\frac{V_{m}+46.463}{8.8289}\right)},$$

$$\alpha_{h}=\frac{\frac{1}{1+\exp\left(\frac{V_{m}+32.2}{4}\right)}}{1.218+42.043\exp\left(-\frac{{\left(V_{m}+38.1\right)}^{2}}{2\cdot 15.19^{2}}\right)},$$

$$\beta_{h}=\frac{1-\frac{1}{1+\exp\left(\frac{V_{m}+32.2}{4}\right)}}{1.218+42.043\exp\left(-\frac{{\left(V_{m}+38.1\right)}^{2}}{2\cdot 15.19^{2}}\right)}.$$

### Quantification of synergy

For each neuron, an additive expectation surface was calculated from the independent current threshold penalties produced by single-axis Nav1.7 or Nav1.8 subtraction. Synergy was defined as the excess observed current threshold shift above this additive prediction at each combinatorial node and was expressed as percent deviation from expected.

### Clustering and computational analysis

To investigate heterogeneity across the Nav1.7/Nav1.8 conductance landscape, each of the 24 neurons was represented by its baseline-normalized 4 × 4 current threshold response matrix, yielding a 16-dimensional feature vector. Spectral clustering grouped neurons according to similarities in their responses across the complete perturbation matrix using a nearest-neighbor affinity graph. The number of clusters was set to three, motivated by the three-component Gaussian mixture model of responses to complete Nav1.8 subtraction. These groups were used as a descriptive framework for examining functional heterogeneity rather than as evidence for discrete neuronal subtypes.

**Figure S2.**
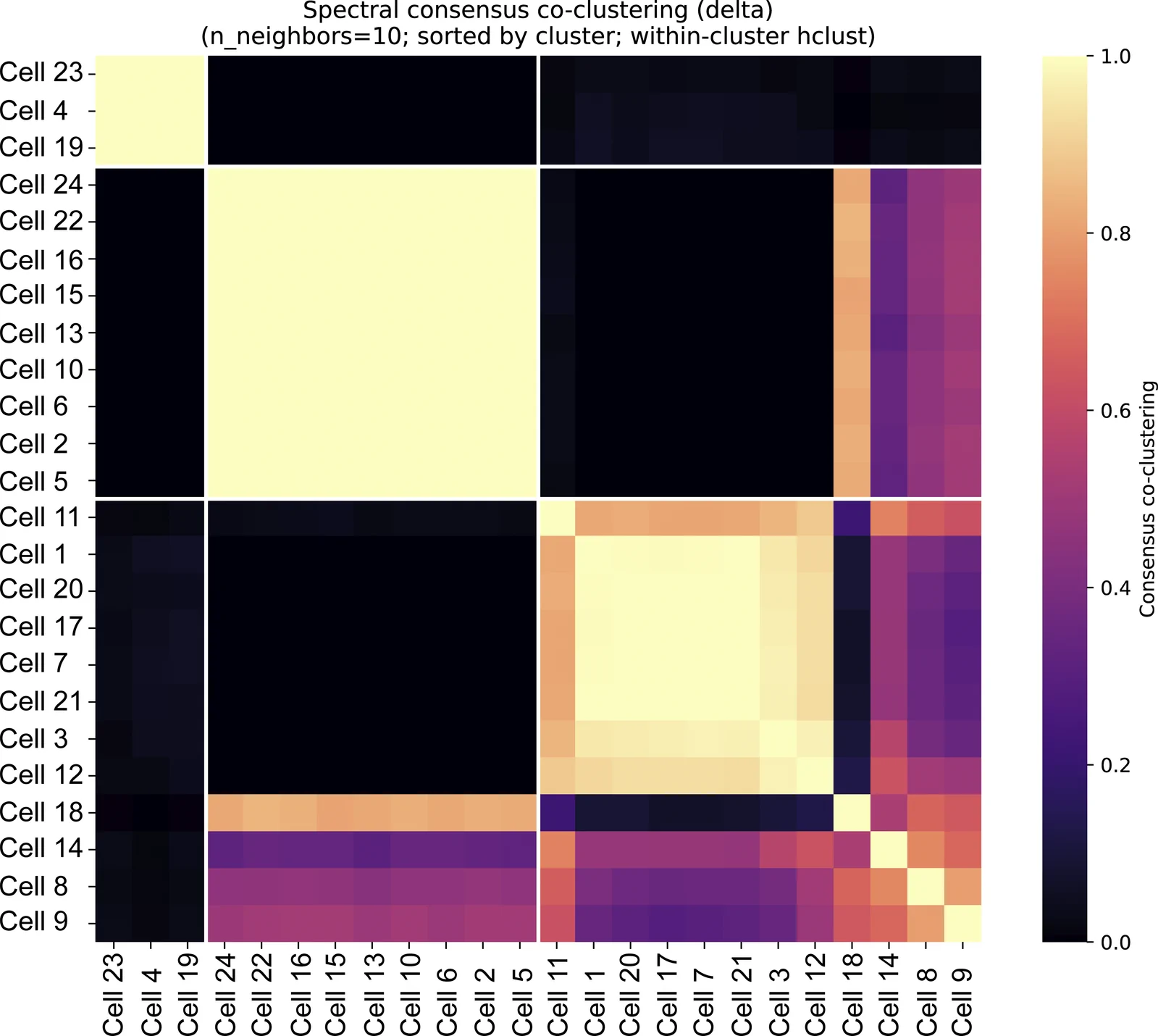
**Consensus co-clustering matrix demonstrating robustness of spectral clustering assignments.** Each row and column represents one neuron. Each matrix entry indicates the fraction of repeated runs of the spectral-clustering nearest-neighbor graph in which a pair of neurons was assigned to the same cluster. Values range from 0 (never assigned together; dark) to 1 (always assigned together; light). Neurons are ordered by their final cluster assignment and hierarchically ordered within each cluster; white lines indicate the boundaries of the three-cluster solution. Light diagonal blocks indicate consistently shared assignments, whereas intermediate values indicate assignments that were less stable across perturbations. Thus, the matrix evaluates the reproducibility of the three-cluster solution within this dataset.

Principal component analysis (PCA) was used to visualize the response vectors in two dimensions, with neurons colored according to their spectral-cluster assignments. Group-average current threshold and synergy surfaces were generated from the original response values. Internal validation indices, repeated random initializations, and consensus co-clustering (Fig. S2) were used to assess the coherence and reproducibility of the clustering solution. Detailed implementation parameters and validation results are provided in the Supplementary text at the end of the PDF.

### Statistics

Data are presented as mean ± SEM unless otherwise noted. Statistical comparisons were performed using appropriate parametric or nonparametric tests depending on distribution and experimental design, with significance set at P < 0.05. See Table 1 for a description and results of all statistical tests.

**Table 1.** Statistical summary

Figure	Test type	Effect or comparison	p^a^
Fig. 2 D	Shapiro–Wilk test	Normality	<0.0001
Fig. 2 D	One-sample Wilcoxon signed-rank test	Average synergy vs. 0	<0.0001
Fig. 4 A	Shapiro–Wilk test	Normality	0.1383
Fig. 4 B	Shapiro–Wilk test	Normality	<0.0001
Fig. 4 E	Kruskal–Wallis test	​	0.5471
Fig. 4 F	Kruskal–Wallis test	​	<0.0001
​	Dunn’s multiple comparison test		​
​	​	Strong vs. weak	0.0004
​	​	Strong vs. Int	0.2931
​	​	Weak vs. Int	0.0021
Fig. 4 G	Kruskal–Wallis test	​	0.1431
Fig. 4 H	Kruskal–Wallis test	​	0.0296
​	Dunn’s multiple comparison test		​
​	​	Strong vs. weak	0.0274
​	​	Strong vs. Int	0.2624
​	​	Weak vs. Int	0.4493
Fig. 4 I	Kruskal–Wallis test	​	0.0027
​	Dunn’s multiple comparison test		​
​	​	Strong vs. weak	0.0140
​	​	Strong vs. Int	>0.9999
​	​	Weak vs. Int	0.0117
Fig. 4 J	Kruskal–Wallis test	​	0.0654
Fig. 4 K	Kruskal–Wallis test	​	0.0004
​	Dunn’s multiple comparison test		​
​	​	Strong vs. weak	0.0130
​	​	Strong vs. Int	>0.9999
​	​	Weak vs. Int	0.0010
Fig. 4 L	Kruskal–Wallis test	​	0.0020
​	Dunn’s multiple comparison test	​	​
​	​	Strong vs. weak	0.0151
​	​	Strong vs. Int	>0.9999
​	​	Weak vs. Int	0.0071
Fig. 4 M	Kruskal–Wallis test	​	0.0350
​	Dunn’s multiple comparison test	​	​
​	​	Strong vs. weak	0.0295
​	​	Strong vs. Int	0.1837
​	​	Weak vs. Int	0.7347
Fig. 4 N	Fisher’s exact test	​	0.4065
Fig. 7 D	Kruskal–Wallis test	​	0.0003
​	Dunn’s multiple comparison test		​
​	​	Strong vs. weak	0.0061
​	​	Strong vs. Int	>0.9999
​	​	Weak vs. Int	0.0011
Fig. 8 E	Kruskal–Wallis test	​	<0.0001
​	Dunn’s multiple comparison test		​
​	​	Strong vs. weak	0.0004
​	​	Strong vs. Int	0.3010
​	​	Weak vs. Int	0.0023
Fig. S4	Linear regression	​	0.0032

a Adjusted P values for multiple comparisons testing.

### Online supplemental material

**Figure S3.**
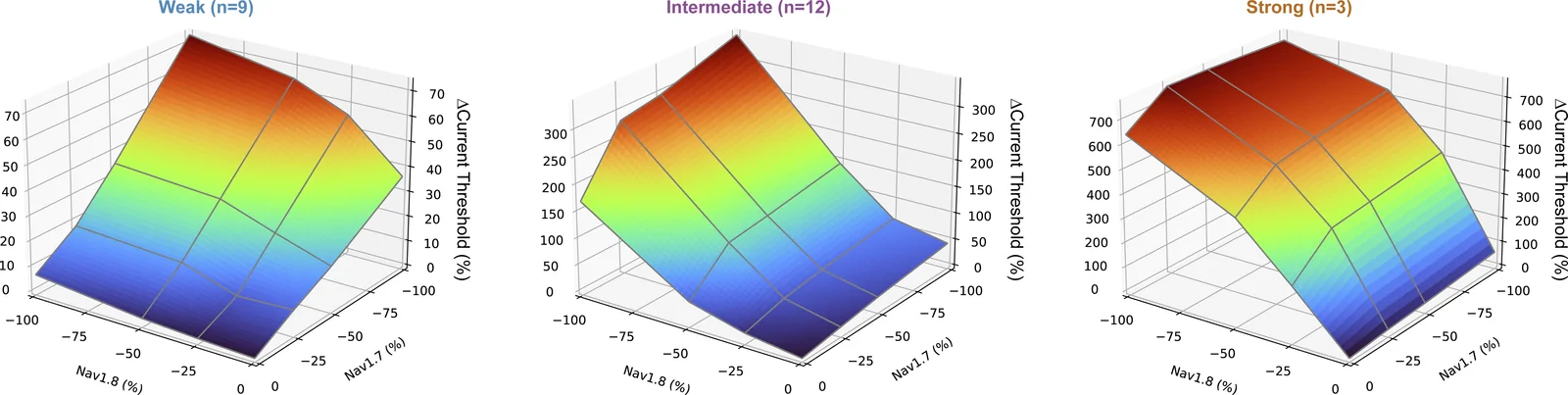
**Current threshold (percentage change from baseline) response surfaces.** Mean 3-D current threshold (percentage change from baseline) response surfaces for weak, intermediate, and strong Nav1.8 response modes across the 16-node Nav1.7 × Nav1.8 functional subtraction matrix.

**Figure S4.**
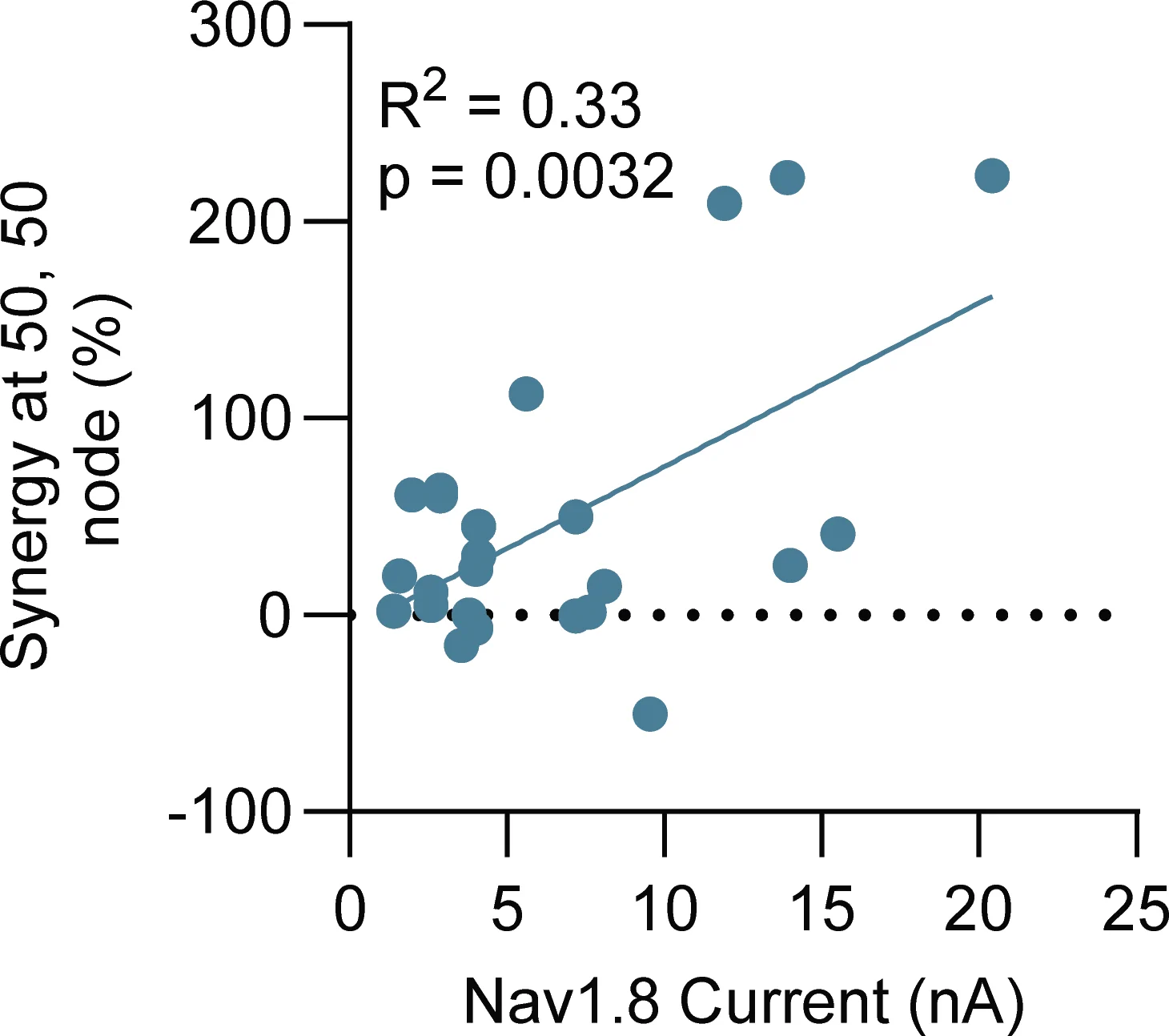
**Linear regression of Nav1.8 current at baseline and synergy at the 50, 50 node.** Nav1.8 current at baseline significantly correlates with the synergy value at the 50, 50 node but only explains 33% of the variation.

**Figure S5.**
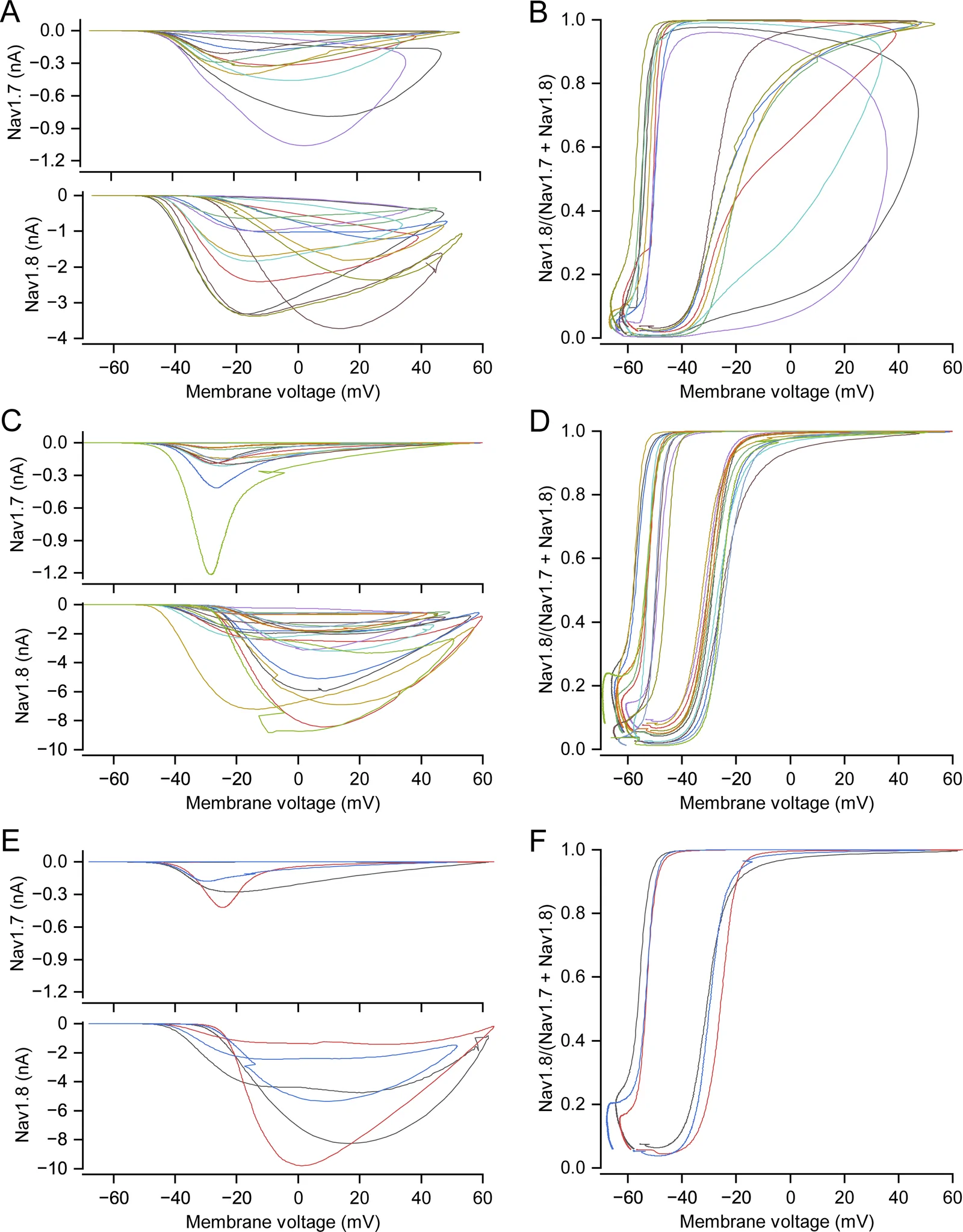
**Individual-cell Nav1.7 and Nav1.8 current trajectories across the Nav1.8 sensitivity spectrum. (A, C, and E)** Nav1.7 (upper panels) and Nav1.8 (lower panels) current trajectories calculated during control APs for individual weak (A), intermediate (C), and strong (E) Nav1.8 responders. Each colored line represents an individual neuron. **(B, D, and F)** Corresponding voltage-dependent trajectories of the relative Nav1.8 contribution, Nav1.8/(Nav1.7 + Nav1.8), for weak (B), intermediate (D), and strong (F) responders. These individual-cell trajectories underlie the response-group averages shown in Fig. 7. Trajectory averages were calculated separately for the outward (depolarizing) and return (repolarizing) branches of each trace, with the turning point defined by the maximum membrane voltage. Within each branch, the first crossing of each Vm in the direction of trajectory evolution was used, ensuring that each cell contributed no more than one value at a given Vm. Individual traces were linearly interpolated to common Vm values, and the corresponding values were then averaged across cells. This procedure preserved the voltage-dependent trajectory and avoided averaging multiple points from the same trace that occurred at the same Vm during different phases of the trajectory (see Fig. 7).

Supplemental text at the end of the PDF includes additional methodological details for the computational and clustering analyses, as well as supplementary analyses and figures supporting response-spectrum and recording-stability assessments. Fig. S1 shows stability of electrophysiological parameters during the dynamic-clamp recording protocol. Fig. S2 shows consensus co-clustering matrix demonstrating robustness of spectral clustering assignments. Fig. S3 shows current threshold (percentage change from baseline) response surfaces. Fig. S4 shows linear regression of Nav1.8 current at baseline and synergy at the 50, 50 node. Fig. S5 shows individual-cell Nav1.7 and Nav1.8 current trajectories across the Nav1.8 sensitivity spectrum.

## Results

To determine whether Nav1.7 and Nav1.8 contribute independently or nonlinearly (synergistically or antagonistically) to AP electrogenesis in DRG neurons, we used dynamic clamp to subtract precisely defined fractions of each conductance while recording neuronal excitability in current-clamp mode. Subtraction of the current attributable to a given channel provides a proxy for block with an idealized agent with optimal pharmacodynamics, pharmacokinetics, and distribution. This approach required very long, stable recordings from each neuron but permitted extensive perturbations to be made in each cell successfully studied and enabled us to move beyond isoform-selective perturbation and experimentally map the combinatorial conductance landscape governing AP initiation. In each neuron, multiple recordings in current-clamp mode were taken, where cells were subjected to a 16-node perturbation matrix in which the two conductances were functionally subtracted in discrete combinations spanning 0, 25, 50, and 100% of each isoform (Fig. 1 A). Current threshold was then measured at each node as a direct readout of the minimal stimulation current required to trigger an AP. Our analysis required 20 current threshold measurements per neuron for a 4 × 4 matrix and 4 control recordings for every matrix column for each cell, requiring a minimum of 40–50 min of stable recordings (stable RMP and current threshold). If a small drift in measured parameters was observed, it was accounted for, as parameter changes within each matrix column were calculated relative to the most recent control recording. We did not perform full-matrix recordings on cells with an AP overshoot below +34 mV (*n* = 9). Approximately 60% of cells met these stability and AP overshoot criteria (*n* = 24 out of 41 neurons recorded). The neurons completing the full perturbation matrix had a mean soma diameter of 25.88 ± 0.23 µm (*n* = 24). At baseline, Nav1.7 and Nav1.8 current amplitudes were 12.1 ± 1.1 nA and 6.7 ± 1.0 nA, respectively, with Nav1.8 accounting for 0.25 ± 0.02 of total sodium current. The population had a mean RMP of −56.9 ± 0.9 mV, voltage threshold of −25.7 ± 1.2 mV, AP width of 2.3 ± 0.1 ms, overshoot of 48.7 ± 1.7 mV, undershoot of −63.9 ± 0.5 mV, input resistance of 176.1 ± 13.3 MΩ, and current threshold of 326.0 ± 34.8 pA (Fig. 1).

### Systematic perturbation mapping reveals synergy between Nav1.7 and Nav1.8 in AP electrogenesis in sensory neurons

**Figure 2.**
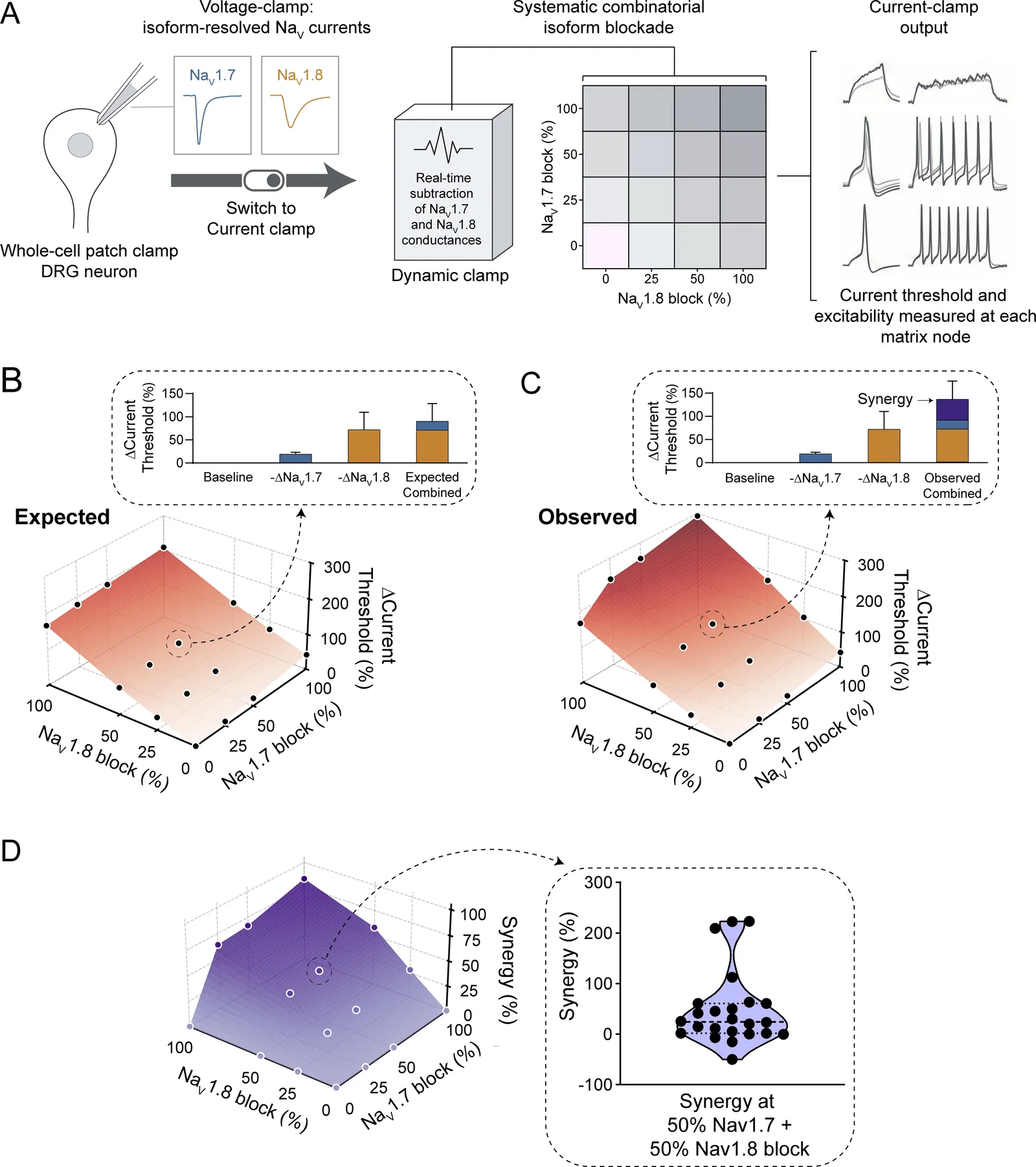
**Systematic mapping of Nav1.7 and Nav1.8 combinatorial interactions via dynamic clamp. (A)** Experimental workflow for dynamic clamp investigation of combinatorial isoform blockade in DRG neurons. Nav1.8 current was isolated using depolarizing voltage steps from a holding potential of −45 mV, at which most other sodium channel isoforms are inactivated while Nav1.8 remains available. Nav1.7 current was estimated from the tetrodotoxin-sensitive sodium current component based on prior evidence that Nav1.7 constitutes ∼70% of tetrodotoxin-sensitive current in small DRG neurons (Vasylyev et al., 2014). Following voltage-clamp characterization of Nav1.7 and Nav1.8 currents, cells are switched to current-clamp mode and successive functional subtraction of specific conductances simulates systematic combinatorial blockade across a 16-node matrix. **(B)** Expected current threshold response across the Nav1.7–Nav1.8 subtraction matrix. The inset shows the mean ± SEM increases in current threshold produced by 50% subtraction of Nav1.7 or Nav1.8 individually and the expected combined response, calculated for each neuron as the sum of the two individual effects. **(C)** Observed current threshold response across the subtraction matrix. The inset shows the corresponding mean ± SEM observed response during combined 50% Nav1.7 and 50% Nav1.8 subtraction; the portion exceeding the expected response represents synergy. **(D)** Mean synergy surface across all neurons, calculated as the observed response minus the expected additive response at each matrix node. The violin plot shows individual-neuron synergy values at the 50% Nav1.7/50% Nav1.8 subtraction node.

We first asked whether the effect of combined Nav1.7 and Nav1.8 attenuation could be predicted by summation of the increase in current threshold produced by the subtraction of each isoform in isolation. For each neuron, we therefore generated an expectation surface in which the current threshold shift at each combinatorial node was calculated from the independent effects of subtracting each isoform alone (Fig. 2 B). Under this framework of independent effects, dual attenuation might have been expected to increase current threshold in a manner that reflects only the arithmetic sum of the two isoform-selective perturbations. However, comparison of these expected surfaces with the actual dynamic clamp recordings revealed a markedly different behavior. In many neurons, the observed current threshold at combinatorial nodes substantially exceeded the independent additive prediction, producing a prominent upward deviation in the response surface that was absent from the corresponding monotherapy axes (Fig. 2 C). Subtraction of either Nav1.7 or Nav1.8 alone produced the expected increase in current threshold, but partial subtraction of both channels together often drove a far larger suppression of excitability than would be anticipated from either perturbation in isolation (observed in 20/24 neurons recorded; Fig. 2 D). In these neurons, the membrane did not behave as though Nav1.7 and Nav1.8 were independent depolarizing reserves. Instead, the effect of attenuating one conductance depended strongly on the remaining availability of the other, indicating that the two channels operate as an electrogenic dyad functionally coupled in a nonlinear manner.

### Cell-to-cell variability of the Nav1.7–Nav1.8 synergistic interaction

**Figure 3.**
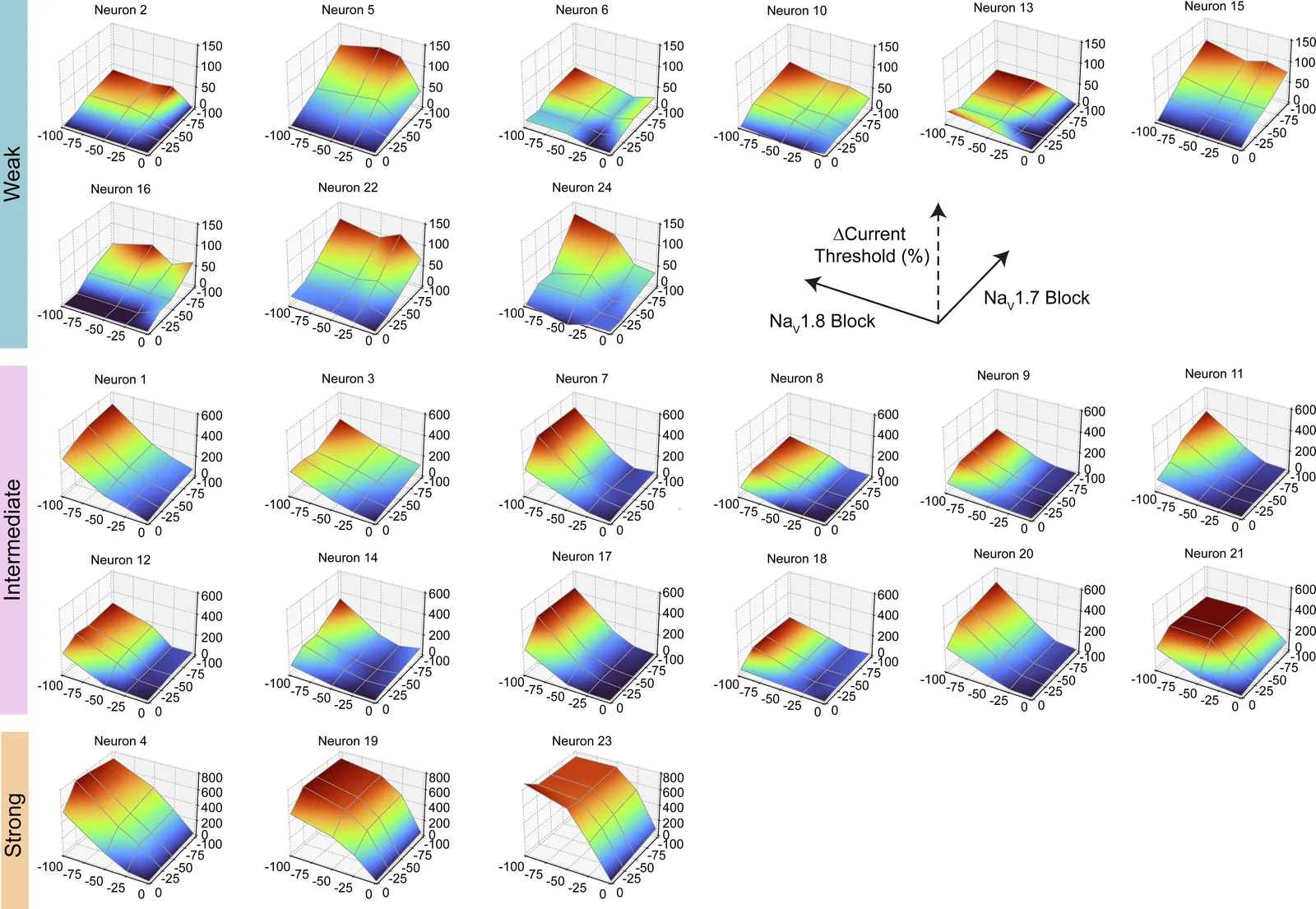
**Individual cell current threshold percentage change from baseline response surfaces; 3-D surfaces mapping percent increases in current threshold across varying percentages of Nav1.7 and Nav1.8 functional subtraction.** Each plot represents recordings from an individual cell. Current threshold change is measured as a percentage change from baseline. Note the difference in surface directionality between cells at different points of the Nav1.8 response spectrum (labeled on the left). For visual presentation, neurons are organized according to the response-mode assignments derived from the distributional and clustering analyses presented in Fig. 4.

To quantify this interaction across the population, we calculated the percent of synergistic interaction at each matrix node, defined as the excess current threshold shift above the linear additive expectation. We focused on the 50/50% subtraction node because it provided a balanced and physiologically informative measure of partial dual attenuation of excitability. At this node, the distribution of synergistic interaction across neurons was shifted above zero, demonstrating that the population response was dominated by supralinear rather than independent linear additive behavior (Synergy averaged 52.1 ± 15.0%, and the distribution was significantly shifted above zero, Wilcoxon signed-rank test, P <0.0001; Fig. 2 D). Notably, the magnitude of this interaction varied substantially between cells (ranging from −50 to 223%), suggesting that although synergy is a general feature of the sensory neuronal system, its strength is not uniform across the population. Current threshold response vectors for each of the cells studied are shown in Fig. 3.

These results demonstrate the nonlinearity of the functional interplay of Nav1.7 and Nav1.8 channels driving DRG neuron excitability, and show that there is substantial cell-to-cell variability of their synergistic interaction. As a consequence of this, the functional effect of jointly attenuating Nav1.7 and Nav1.8 cannot be inferred from single-isoform perturbations alone. Instead, partial reduction of both conductances produces a supralinear increase in current threshold in a substantial fraction of sensory neurons.

### Sensitivity to Nav1.8 functional subtraction spans a broad response spectrum

**Figure 4.**
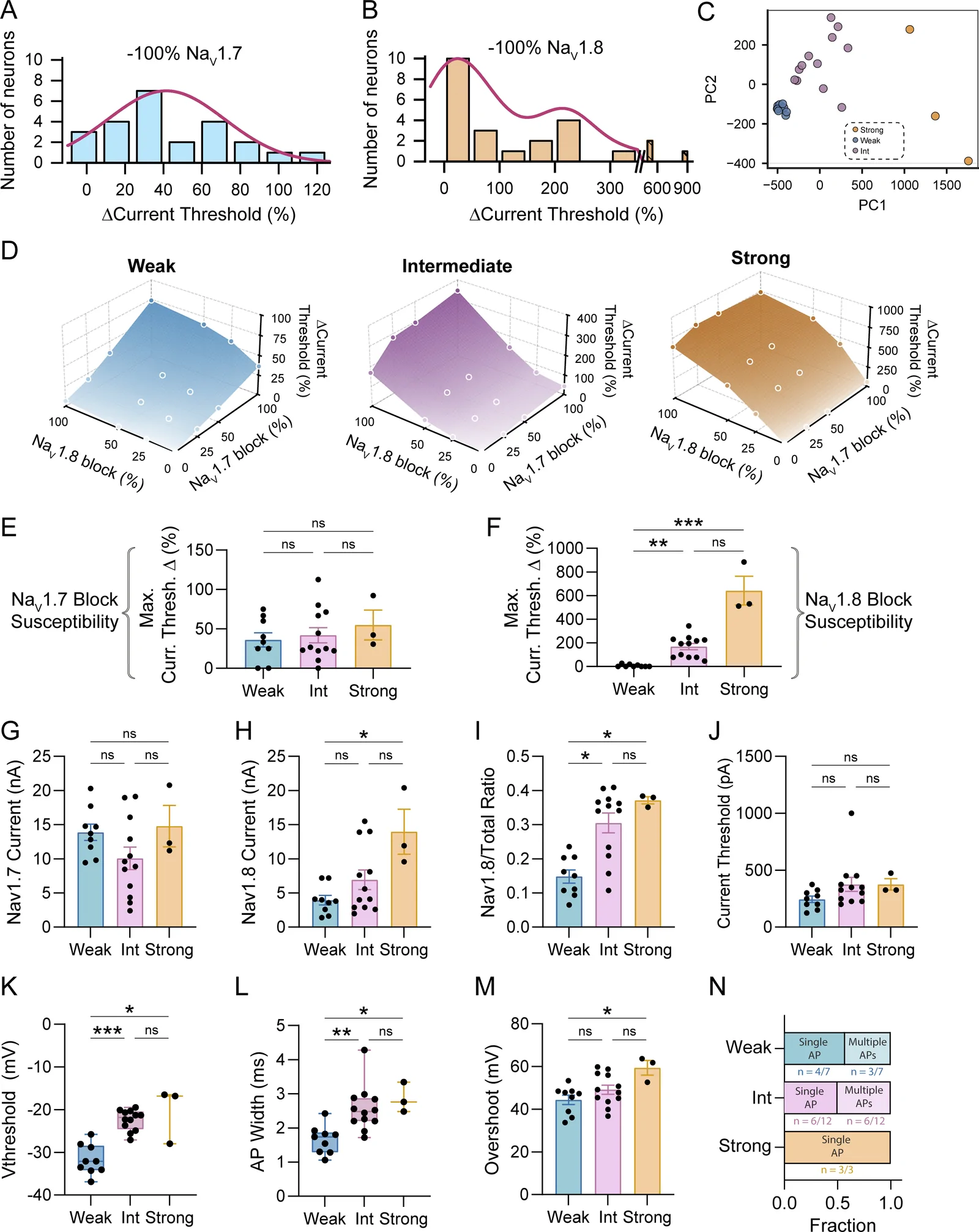
**Sensitivity to Nav1.8 functional subtraction spans a broad response spectrum. (A and B)** Distribution of maximum current threshold changes produced by complete functional subtraction of Nav1.7 (A) or Nav1.8 (B) across recorded neurons. Current threshold change is expressed as percentage change from baseline. Responses to Nav1.7 subtraction were comparatively constrained, whereas responses to Nav1.8 subtraction spanned a broad range. Gaussian mixture modeling of responses to complete Nav1.8 subtraction supported weak, intermediate, and strong response modes. These modes were used as a descriptive framework for examining neurons occupying different regions of the broader Nav1.8 sensitivity spectrum. **(C)** Unsupervised spectral clustering of the 16-node response vectors shows the organization of the three-group spectral-clustering solution in PCA space (weak, intermediate, strong responders). **(D)** Mean 3-D current threshold response surfaces for weak, intermediate, and strong Nav1.8 response modes across the 16-node Nav1.7 × Nav1.8 functional subtraction matrix. Note the different z-axis scales. Surfaces show the percentage change in current threshold relative to baseline, with color scales adjusted to each group’s response range. Group-specific hues preserve the identification of the weak-, intermediate-, and strong-response groups, while color intensity represents Δcurrent threshold within each group’s displayed range. Fig. S3 presents Δcurrent threshold using a common color scheme to facilitate comparison across groups. **(E and F)** Maximum current threshold change produced by Nav1.7 functional subtraction (E) or Nav1.8 functional subtraction (F) in weak, intermediate, and strong Nav1.8 responders. **(G–I)** Baseline sodium current properties across the Nav1.8 response spectrum: isolated Nav1.7 current (G), isolated Nav1.8 current (H), and the ratio of Nav1.8 to total sodium current (I). **(J–M)** Baseline electrophysiological properties across weak, intermediate, and strong Nav1.8 response modes: current threshold (J), AP voltage threshold (K), AP width (L), and AP overshoot (M). **(N)** Proportion of neurons within each response mode exhibiting single or multiple APs during a 500-ms depolarizing current injection. Bars show mean ± SEM and points represent individual neurons. Statistical comparisons in panels E–M were performed using Kruskal–Wallis tests followed, where appropriate, by Dunn’s multiple-comparisons tests. Firing-pattern distributions in panel N were compared using Fisher’s exact test. *P < 0.05, **P < 0.01, ***P < 0.001; ns, not significant.

The cell-to-cell variability in synergistic interaction and in current threshold response matrix observed in Fig. 2 and Fig. 3 suggested that the combinatorial logic of the Nav1.7–Nav1.8 dyad is not uniform across the DRG neuronal population. Importantly, using the same methods for primary DRG neuron culture and dynamic clamp recordings, we previously identified strong- and weak-responder DRG neurons based on their sensitivity to Nav1.8 functional subtraction (Vasylyev et al., 2025). To assess the minimal number of cells required to probe current threshold sensitivity within a two-dimensional conductance-perturbation matrix incorporating both Nav1.7 and Nav1.8, we doubled the sample size (*n* = 24 in this study) to account for the additional dimension. We first examined the distribution of current threshold responses to complete functional subtraction of Nav1.7 or Nav1.8 across the 24 neurons studied (Fig. 4, A and B). Responses to complete (100%) Nav1.7 subtraction were normally distributed (P = 0.14 by the Shapiro–Wilk test), and maximum current threshold increases averaged 41.4 ± 6.2% (range: 0–113%; Fig. 4 A) across neurons. In contrast, responses to complete Nav1.8 abrogation varied by nearly an order of magnitude more, ranging from 0 to 885%. The distribution of responses to Nav1.8 subtraction was strongly right-skewed (Fig. 4 B; skewness = 1.91) and deviated significantly from normality (Shapiro–Wilk, P < 0.0001), reflecting substantial heterogeneity in the contribution of Nav1.8 to AP initiation across the sampled population. The marked heterogeneity in responses to complete Nav1.8 subtraction prompted formal assessment of the underlying response distribution.

Gaussian mixture modeling was performed to distinguish continuous right-skew from latent response modes. Bayesian Information Criterion (BIC) favored progressively more complex models, with BIC values of 331.4, 309.2, and 301.5 for one-, two-, and three-component models, respectively. The three-component model therefore provided the best overall fit (ΔBIC = 29.9 versus one component; ΔBIC = 7.7 versus two components), supporting weak-, intermediate-, and strong-response modes within the distribution of current threshold responses to complete Nav1.8 subtraction. Visual inspection of the histogram and kernel density estimate was consistent with the formal distributional analyses. Responses to complete Nav1.7 subtraction exhibited a single principal mode, whereas responses to complete Nav1.8 subtraction exhibited two principal modes corresponding to weak and intermediate responses, together with a small group of three highly responsive cells (Fig. 4, A and B). The Nav1.8 response distribution therefore contained at least two dominant modes, together with a sparsely sampled high-response mode. Despite the modest sample size (*n* = 24), convergent evidence from non-normality testing and mixture-model selection supports the presence of latent functional response modes within the sampled neuronal population, although these analyses alone do not establish discrete sensory neuron subtypes.

Collectively, these findings demonstrate that neuronal current threshold modulation was not uniformly distributed across the sampled population, but instead exhibited response modes that became increasingly apparent with stronger Nav1.8 perturbation. Isolated Nav1.7 suppression remained comparatively homogeneous, whereas conditions involving strong or complete Nav1.8 suppression consistently revealed non-normal, multimodal structure. These findings identify Nav1.8 sensitivity as a dominant axis of functional heterogeneity, while Nav1.7 appears to provide a comparatively uniform and cooperative contribution. The emergence of distinct response modes under maximal Nav1.8 perturbation may reflect differences in recruitment threshold, intrinsic membrane properties, or subtype-associated channel dependencies. These observations motivated multidimensional clustering of the complete 16-node response matrices to determine whether the response modes identified along the Nav1.8 axis were also reflected in the overall topology of the Nav1.7–Nav1.8 response landscape.

To establish a descriptive framework for analyzing this continuous heterogeneity across the full matrix, we applied spectral clustering to each neuron’s complete baseline-normalized 16-node current threshold response vector. The number of clusters was set to three, motivated by the three-component Gaussian mixture model of responses to complete Nav1.8 subtraction. Spectral clustering assigned 9, 12, and 3 neurons to the three groups. For visualization, the 16-dimensional response vectors were projected onto the first two principal components and colored according to their spectral-cluster assignments (Fig. 4 C). The organization of the neurons in PCA space was consistent with weak-, intermediate-, and strong-response patterns extending across the complete perturbation matrix rather than being determined solely by the response at the 100% Nav1.8-subtraction node.

### Nav1.8 dependence is a dominant contributor to functional heterogeneity between neurons

Nav1.8 sensitivity is associated with differences in baseline electrogenic properties. Given the modest sample size (which is a function of the complexity involved in acquiring these recordings and the resultant requirement for at least 40–50 min of stable recording for each cell), we use the weak, intermediate, and strong response modes only as an operational, descriptive framework for examining neurons across the broader Nav1.8 sensitivity spectrum. These categories should not be interpreted as demonstrating the presence of discrete neuronal populations, and the observed heterogeneity may instead reflect functional variation along a continuum. Nevertheless, irrespective of the underlying distribution, the sampled neurons included both weak or nonresponders and strong responders to Nav1.8 subtraction. Mean current threshold response surfaces illustrate the progressive increase in sensitivity across weakly, intermediately, and strongly responsive neurons (Fig. 4 D and Fig. S3). Maximal responses to Nav1.7 subtraction did not differ significantly across these response modes (36.11 ± 9.05%, 42.02 ± 9.67%, and 55.06 ± 18.91% for weak, intermediate, and strongly responsive neurons, respectively; P = 0.55, Kruskal–Wallis test; Fig. 4 E), whereas maximal responses to Nav1.8 subtraction differed markedly (P < 0.0001, Kruskal–Wallis test; Fig. 4 F). Weak responders exhibited minimal current threshold changes following Nav1.8 subtraction (6.6 ± 3.5%, *n* = 9), intermediate responders showed substantially larger responses (169.0 ± 26.3%, *n* = 12), and strong responders showed the largest increases (642.0 ± 121.5%, *n* = 3).

These findings identify Nav1.8 dependence as a dominant axis of functional heterogeneity across the sampled neuronal population, whereas the contribution of Nav1.7 is comparatively uniform and acts primarily as a cooperative factor whose attenuation can amplify the effects of Nav1.8 functional loss.

Sensitivity to Nav1.8 subtraction reflects broad electrogenic context rather than channel abundance alone.

We next asked whether sensitivity to Nav1.8 subtraction was associated with differences in baseline sodium current properties. Nav1.7 current amplitude showed no clear relationship with position along the Nav1.8 response spectrum (13.88 ± 1.18 nA, 10.06 ± 1.65 nA, and 14.77 ± 3.03 nA for weak, intermediate, and strongly responsive neurons, respectively; P = 0.14, Kruskal–Wallis test; Fig. 4 G), whereas isolated Nav1.8 current (3.94 ± 0.70 nA, 6.93 ± 1.43 nA, and 13.95 ± 3.29 nA for weak, intermediate, and strongly responsive neurons, respectively; P = 0.03, Kruskal–Wallis test; post-hoc tests: P = 0.0274 for strong vs. weak, P = 0.2624 for strong vs. int, P = 0.4493 for weak vs. int; Fig. 4 H) and the Nav1.8-to-total sodium current ratio (0.15 ± 0.02, 0.31 ± 0.03, and 0.37 ± 0.01 for weak, intermediate, and strongly responsive neurons, respectively; P = 0.003, Kruskal–Wallis test; post-hoc tests: P = 0.014 for strong vs. weak, P >0.99 for strong vs. int, P = 0.011 for weak vs. int; Fig. 4 I) differed more clearly between groups. These relationships are consistent with the idea that Nav1.8-rich neurons are more vulnerable to perturbation of the dyad. However, baseline Nav1.8 current alone did not fully determine the response to conductance subtraction. Consistent with this, baseline Nav1.8 current explained only 33% of the variance in synergy at the 50% Nav1.7 + 50% Nav1.8 subtraction node (R^2^ = 0.33, P = 0.003, simple linear regression; Fig. S4). This is important because it indicates that the functional response spectrum reflects integrated electrogenic architecture rather than a simple scalar continuum of Nav1.8 functional expression. In other words, cells with different response phenotypes are not merely “high Nav1.8 expression” versus “lower Nav1.8” cells. Instead, variability in Nav1.8 sensitivity appears to reflect the broader electrogenic context in which Nav1.8 operates rather than channel abundance alone.

### AP properties and firing behavior reflect the underlying electrogenic context

Baseline electrophysiological properties also varied across the Nav1.8 response spectrum. Baseline current threshold did not differ significantly across response modes (244.4 ± 27.6 pA, 375.0 ± 61.7 pA, and 375.0 ± 50.0 pA for weak, intermediate, and strongly responsive neurons, respectively, P = 0.07, Kruskal–Wallis test; Fig. 4 J). However, voltage threshold (−31.7 ± 1.2 mV, −22.4 ± 0.7 mV, and −20.4 ± 3.8 mV for weak, intermediate, and strongly responsive neurons, respectively; P = 0.0004, Kruskal–Wallis test, post-hoc tests: P = 0.013 for strong vs. weak, P > 0.99 for strong vs. int, P = 0.001 for weak vs. int; Fig. 4 K), as well as AP width (1.65 ± 0.14 ms, 2.57 ± 0.19 ms, and 2.86 ± 0.25 ms for weak, intermediate, and strongly responsive neurons, respectively; P = 0.002, Kruskal–Wallis test; post-hoc tests: P = 0.015 for strong vs. weak, P >0.99 for strong vs. int, P = 0.007 for weak vs. int; Fig. 4 L) and overshoot (44.4 ± 2.2 mV, 49.2 ± 2.2 mV, and 59.4 ± 3.4 mV for weak, intermediate, and strongly responsive neurons, respectively; P = 0.04, Kruskal–Wallis test; post hoc tests: P = 0.03 for strong vs. weak, P = 0.18 for strong vs. int, P = 0.73 for weak vs. int; Fig. 4 M) did differ significantly. By contrast, the proportion of neurons exhibiting single versus multiple APs did not differ significantly across the spectrum (P = 0.41, Fisher’s exact test; Fig. 4 N), suggesting that firing pattern alone was insufficient to distinguish neuronal response phenotypes. Interestingly, although the sample size is too small to show statistical significance, all neurons in the strong-response group exhibited only a single AP.

**Figure 5.**
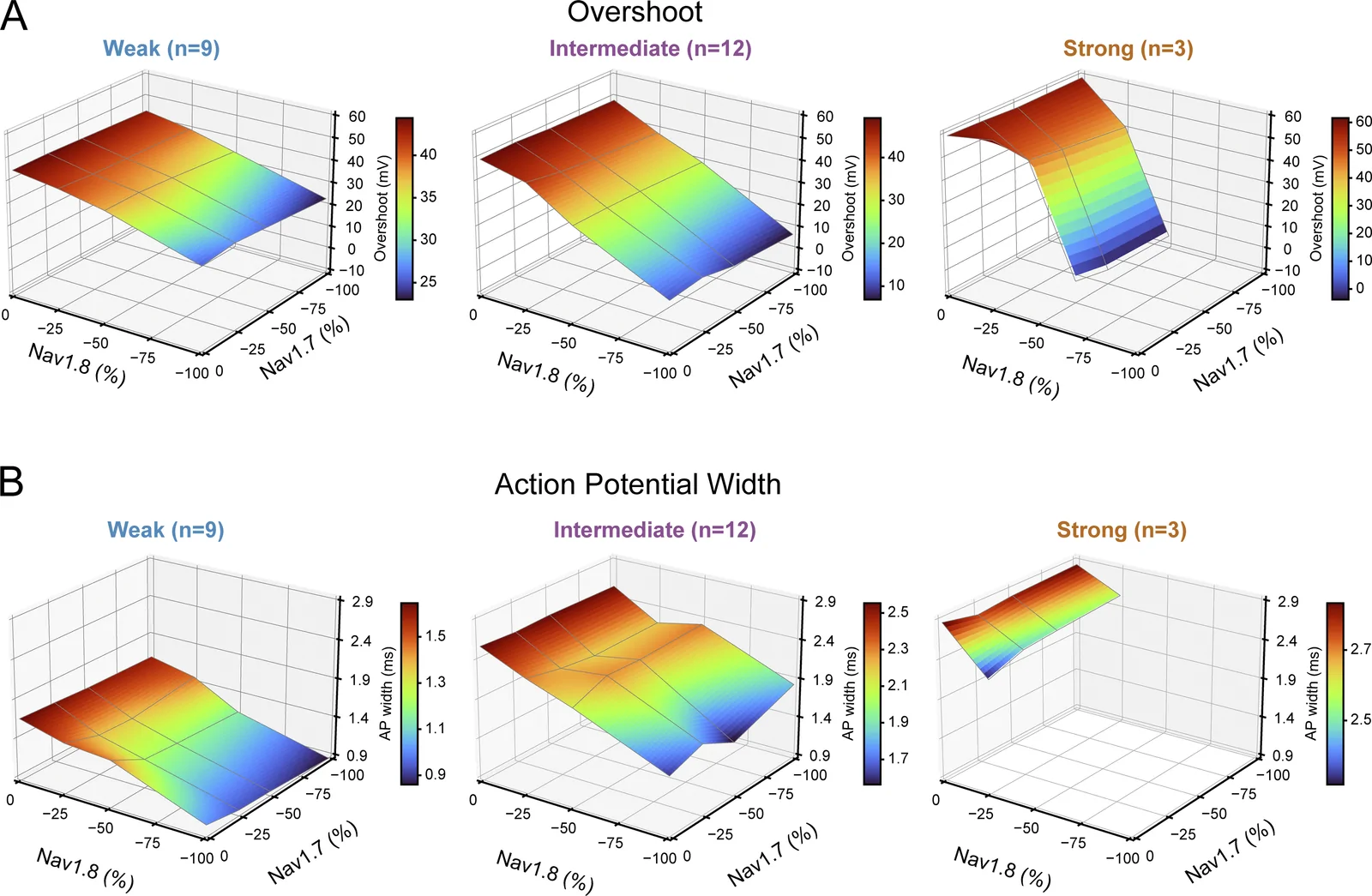
**AP overshoot and AP width vary across the Nav1.8 sensitivity spectrum during conductance subtraction. (A and B)** Mean 3-D surfaces show AP overshoot (A) and AP width (measured at 0 mV, not measured if overshoot was below 0 mV) (B) across combinations of Nav1.7 and Nav1.8 functional subtraction in weak (left panels), intermediate (middle panels), and strong (right panels) Nav1.8 responders. Values within each matrix node represent the mean for neurons within the indicated response mode. Strong Nav1.8 responders exhibited the largest changes in AP overshoot with increasing Nav1.8 and combined Nav1.7/Nav1.8 subtraction, whereas weak responders were comparatively resistant and intermediate responders showed an intermediate response. AP width generally decreased with increasing Nav1.8 functional subtraction, with the magnitude and starting waveform differing across the Nav1.8 sensitivity spectrum. AP width was measured at 0 mV. Blank nodes indicate conditions in which the AP peak did not exceed 0 mV and width was therefore undefined.

These changes were also reflected in the response surfaces of AP morphology parameters. Strong- and intermediate-responsive neurons demonstrated larger reductions in overshoot with combined subtraction than weak-responsive neurons (59.4 to −5.7 mV, 49.2 to 6.4 mV, and 44.4 to 22.7 mV for strong, intermediate, and weak responders, respectively; Fig. 5 A). AP width similarly decreased with combined subtraction among conditions in which APs could still be elicited with AP overshoot above 0 mV (2.86 to 2.56 ms, 2.56 to 1.82 ms, and 1.65 to 0.85 ms for strong, intermediate, and weak responders, respectively; Fig. 5 B). Together, these observations indicate that the effects of Nav1.7–Nav1.8 subtraction reflect the integrated electrophysiological phenotype (including input resistance, RMP, and background conductances) of the cell rather than any single conductance measurement in isolation. Thus, AP waveform shape and firing behavior report the broader electrogenic context in which the functional interplay between Nav1.7 and Nav1.8 is embedded.

**Figure 6.**
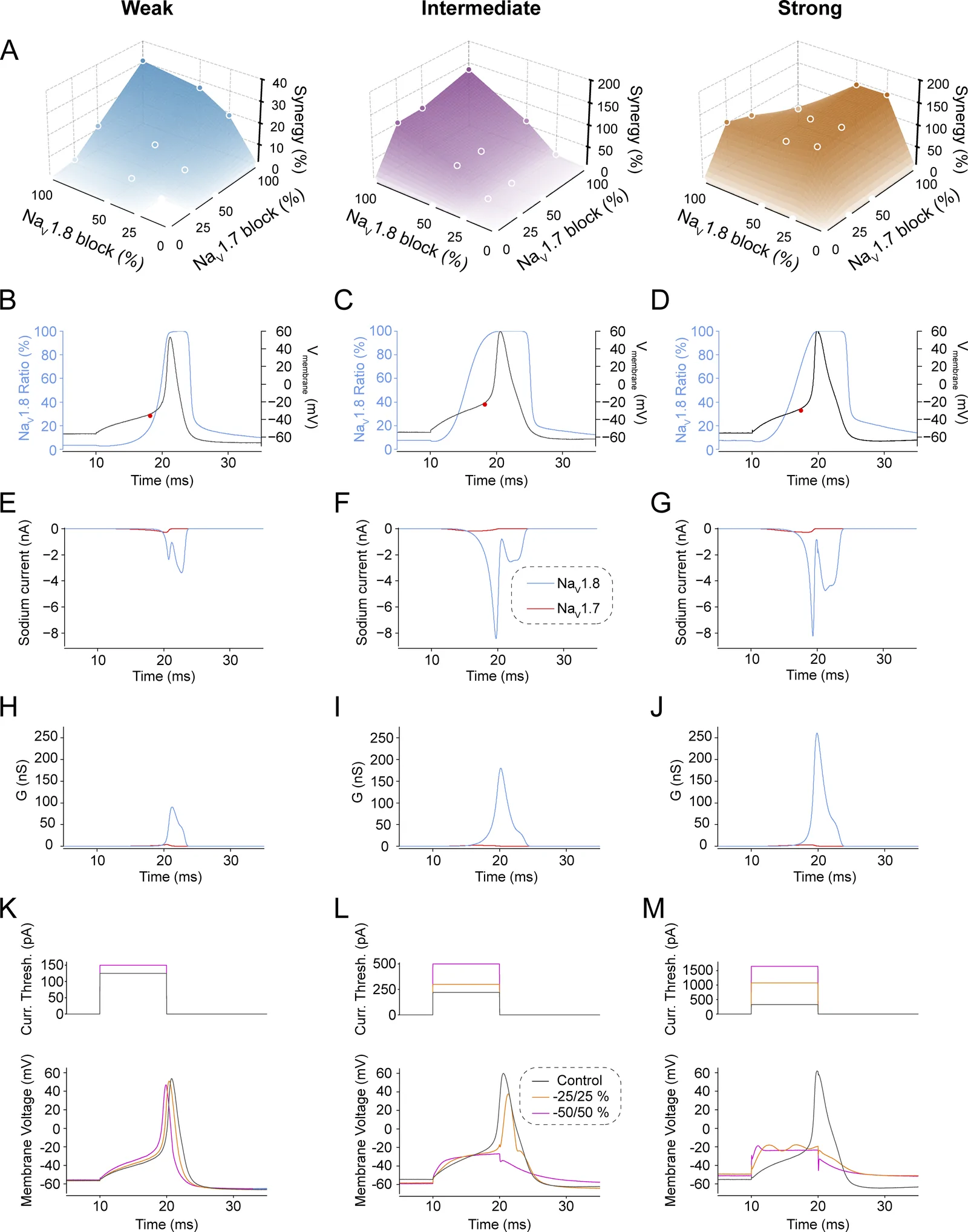
**AP electrogenesis and synergistic susceptibility vary across the spectrum of Nav1.8 sensitivity. (A)** Mean 3-D surfaces depicting synergy across the Nav1.7 × Nav1.8 functional subtraction matrix for weak, intermediate, and strong Nav1.8 response modes. Synergy was defined as the excess observed current threshold increase above the linear additive expectation. **(B–D)** Representative APs from neurons selected from the weak (B), intermediate (C), and strong (D) Nav1.8 response modes. APs were evoked by 10-ms current injections at the current threshold. Red dots indicate the voltage threshold for AP initiation. Blue traces and the left y-axis indicate the relative contribution of Nav1.8 to the combined Nav1.7 and Nav1.8 current calculated throughout the AP. **(E–G)** Nav1.7 (red) and Nav1.8 (blue) sodium currents calculated using an in silico AP clamp for the representative weak (E), intermediate (F), and strong (G) responders shown in B–D. **(H–J)** Corresponding Nav1.7 (red) and Nav1.8 (blue) conductances calculated for the representative weak (H), intermediate (I), and strong (J) responders. Sodium currents were modeled using Hodgkin–Huxley equations and calculated post-hoc from the recorded AP waveforms using APSIM. The fitted maximal conductances were Nav1.7 Gmax = 736 nS and Nav1.8 Gmax = 277 nS for the weak responder, Nav1.7 Gmax = 800 nS and Nav1.8 Gmax = 441 nS for the intermediate responder, and Nav1.7 Gmax = 1,034 nS and Nav1.8 Gmax = 642 nS for the strong responder. **(K–M)** Current-clamp recordings illustrating the effect of progressive combined Nav1.7 and Nav1.8 functional subtraction along the principal diagonal of the perturbation matrix in the same representative weak (K), intermediate (L), and strong (M) responders. Upper panels show the current threshold stimulus and lower panels show the corresponding membrane voltage response under control conditions (black), 25% Nav1.7 + 25% Nav1.8 subtraction (orange), and 50% Nav1.7 + 50% Nav1.8 subtraction (magenta).

AP electrogenesis varies across the Nav1.8 sensitivity spectrum during combined Nav1.7–Nav1.8 attenuation. We next asked whether the supralinear interaction identified prior varied across the spectrum of Nav1.8 sensitivity. Mean synergy surfaces showed a progressive increase in the magnitude of interaction across weak-, intermediate-, and strong-response modes (Fig. 6 A). Weak responders displayed a relatively flat synergy surface, whereas intermediate and strong responders exhibited progressively more prominent synergy peaks within the interior of the matrix, indicating that partial simultaneous subtraction of Nav1.7 and Nav1.8 produced disproportionately larger suppression of excitability in neurons with greater Nav1.8 sensitivity.

### Nav1.8 sensitivity is a dominant contributor to sensory neuron excitability, while Nav1.7 acts as a cooperative conductance

We previously showed that current threshold penalties resulting from Nav1.8 abrogation in nociceptive neurons are correlated with the relative functional contributions of Nav1.7 and Nav1.8 in the perithreshold region (Vasylyev et al., 2025). To examine the biophysical basis of the response spectrum observed here, we compared representative neurons selected from the weak, intermediate, and strong Nav1.8 response modes. These results were derived from Nav1.7 and Nav1.8 Hodgkin–Huxley models in AP clamp mode, using APs obtained from whole-cell dynamic clamp recordings in respective neurons (Fig. 6, B–D). The weak responder exhibited a relatively hyperpolarized voltage threshold of −34 mV and a low (18.6% of the net current) contribution of Nav1.8 relative to Nav1.7 in the perithreshold voltage range. By contrast, the intermediate (93.1% at Vth = −21.4 mV) and strong (68.8% at Vth = −27.9 mV) responders exhibited greater Nav1.8 contributions near AP threshold (Fig. 6, E–J). Consistent with these differences, the current threshold penalty produced by progressive combined attenuation of Nav1.7 and Nav1.8 differed markedly across the three representative neurons. Along the principal diagonal of the Nav1.7–Nav1.8 perturbation matrix, the weak responder showed only a modest increase in current threshold with 25%/25% and 50%/50% functional subtraction, whereas the intermediate and strong responders exhibited progressively larger current threshold penalties (Fig. 6, K–M).

Thus, Nav1.7–Nav1.8 functional synergy is not a binary property that is simply present or absent across the population but varies across a broad response spectrum. Neurons with greater functional dependence on Nav1.8 exhibit a larger contribution of Nav1.8 near AP threshold and a correspondingly greater response to combined Nav1.7 and Nav1.8 attenuation. These findings support a model in which Nav1.8 sensitivity represents a dominant axis of heterogeneity in sensory neuron excitability, while Nav1.7 acts as a cooperative conductance whose attenuation can amplify the consequences of Nav1.8 functional loss.

### Nav1.8 contribution near AP voltage threshold differs across the Nav1.8 sensitivity spectrum

**Figure 7.**
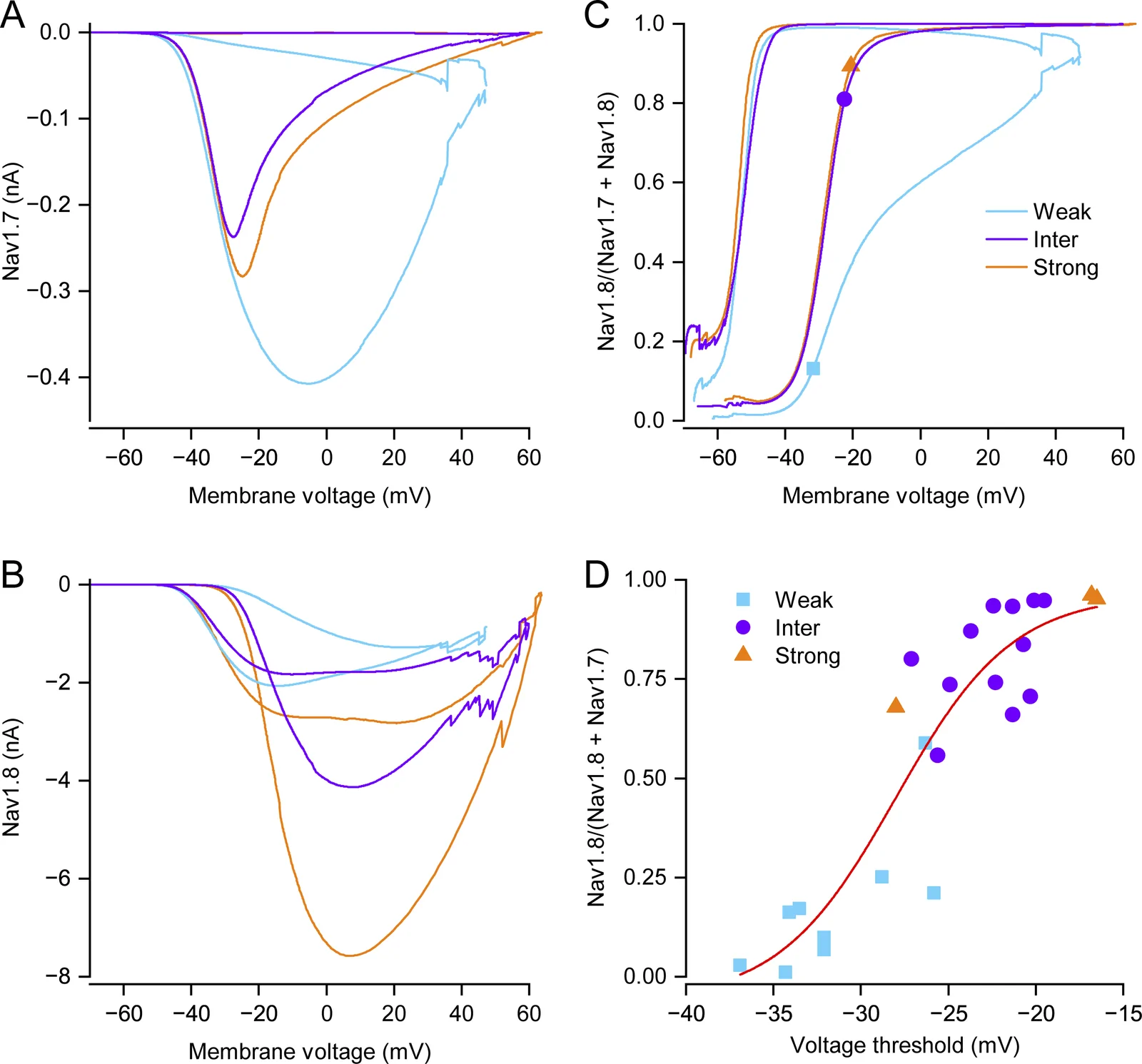
**Nav1.7 and Nav1.8 current trajectories differ across the Nav1.8 sensitivity spectrum. (A)** Mean Nav1.7 current trajectories plotted as a function of membrane voltage for weak (cyan), intermediate (purple), and strong (orange) Nav1.8 responders. **(B)** Mean Nav1.8 current trajectories for the same three response groups. **(C)** Mean voltage-dependent trajectories of the relative Nav1.8 contribution to the combined sodium current, calculated as Nav1.8/(Nav1.7 + Nav1.8). Depolarizing and repolarizing trajectory branches were averaged separately. Symbols indicate the group means at the corresponding mean AP voltage threshold. **(D)** Nav1.8/(Nav1.7 + Nav1.8) at the experimentally measured AP voltage threshold plotted against voltage threshold for individual neurons (*n* = 24). Weak, intermediate, and strong responders are indicated by distinct symbols. The red line represents a four-parameter Boltzmann fit, y = A2 + (A1 − A2)/[1 + exp((x − x0)/dx)], with A1 = −0.0611 ± 0.2431, A2 = 0.9635 ± 0.1416, ×0 = −27.99 ± 1.83 mV, and dx = 3.34 ± 1.98 mV (estimate ± SEM). The fit yielded R^2^ = 0.8313 (adjusted R^2^ = 0.8060). Group comparison: Kruskal–Wallis H = 16.28, P = 0.0003; Dunn’s adjusted P = 0.0011 for weak vs. intermediate, P = 0.0061 for weak vs. strong, and P > 0.9999 for intermediate vs. strong. Across all neurons, Spearman r = 0.890, P < 0.0001.

To determine whether the response modes identified from the Nav1.7–Nav1.8 perturbation matrix were associated with systematic differences in sodium-current recruitment during AP electrogenesis, we calculated Nav1.7 and Nav1.8 current trajectories during control APs for neurons classified as weak, intermediate, or strong Nav1.8 responders. Population trajectories were averaged as a function of membrane voltage separately for the depolarizing and repolarizing phases of the AP (Fig. 7, A–C), while the corresponding individual cell trajectories are shown in Fig. S5. The averaged trajectories revealed a progressive shift in the relative contribution of Nav1.8 to the combined Nav1.7 + Nav1.8 sodium current across the response spectrum.

At the experimentally determined AP voltage threshold, Nav1.8/(Nav1.7 + Nav1.8) was 0.176 ± 0.058 in weak responders (*n* = 9), 0.806 ± 0.037 in intermediate responders (*n* = 12), and 0.864 ± 0.092 in strong responders (*n* = 3; mean ± SEM; Fig. 7 D). The distributions differed significantly across the three groups (Kruskal–Wallis test, H = 16.28, P = 0.0003). Dunn’s multiple-comparison test with adjustment for three pairwise comparisons showed significant differences between weak and intermediate responders (adjusted P = 0.0011) and between weak and strong responders (adjusted P = 0.0061), whereas intermediate and strong responders did not differ significantly (adjusted P > 0.9999). Across all 24 neurons, the Nav1.8 contribution at the threshold was strongly correlated with the voltage threshold (Spearman *r* = 0.890, P < 0.0001), such that neurons with more depolarized thresholds generally exhibited a larger Nav1.8 contribution. Thus, the functional response spectrum is accompanied by a graded difference in the relative recruitment of Nav1.8 in the perithreshold voltage range.

The relationship between AP voltage threshold and the relative Nav1.8 contribution at threshold, Nav1.8/(Nav1.7 + Nav1.8), was well described by a four-parameter Boltzmann function (Fig. 7 D). The fit yielded a lower asymptote of −0.0611 ± 0.2431, an upper asymptote of 0.9635 ± 0.1416, a midpoint of −27.99 ± 1.83 mV, and a slope parameter of 3.34 ± 1.98 mV (parameter estimate ± SE; *n* = 24). The model accounted for 83.1% of the variance (R^2^ = 0.8313; adjusted R^2^ = 0.8060), indicating a steep transition in the relative contribution of Nav1.8 over the perithreshold voltage range. Thus, neurons with more hyperpolarized voltage thresholds were predominantly Nav1.7-dependent at threshold, whereas Nav1.8 became the dominant component as voltage threshold shifted to more depolarized values.

These population data extend the representative-cell analysis by showing that the functional response spectrum is accompanied by a systematic shift in the relative recruitment of Nav1.7 and Nav1.8 during AP initiation. Weak responders reach the threshold while Nav1.7 remains the dominant sodium-current component, whereas intermediate and strong responders generally reach the threshold after substantial recruitment of Nav1.8. Thus, sensitivity to Nav1.8 functional subtraction is associated with the voltage-dependent contribution of Nav1.8 in the perithreshold region rather than simply with the absolute abundance of either sodium-current component.

### Nonlinear interaction boundaries reveal dose-sparing efficacy and an intrinsic limit of dual Nav1.7–Nav1.8 targeting

**Figure 8.**
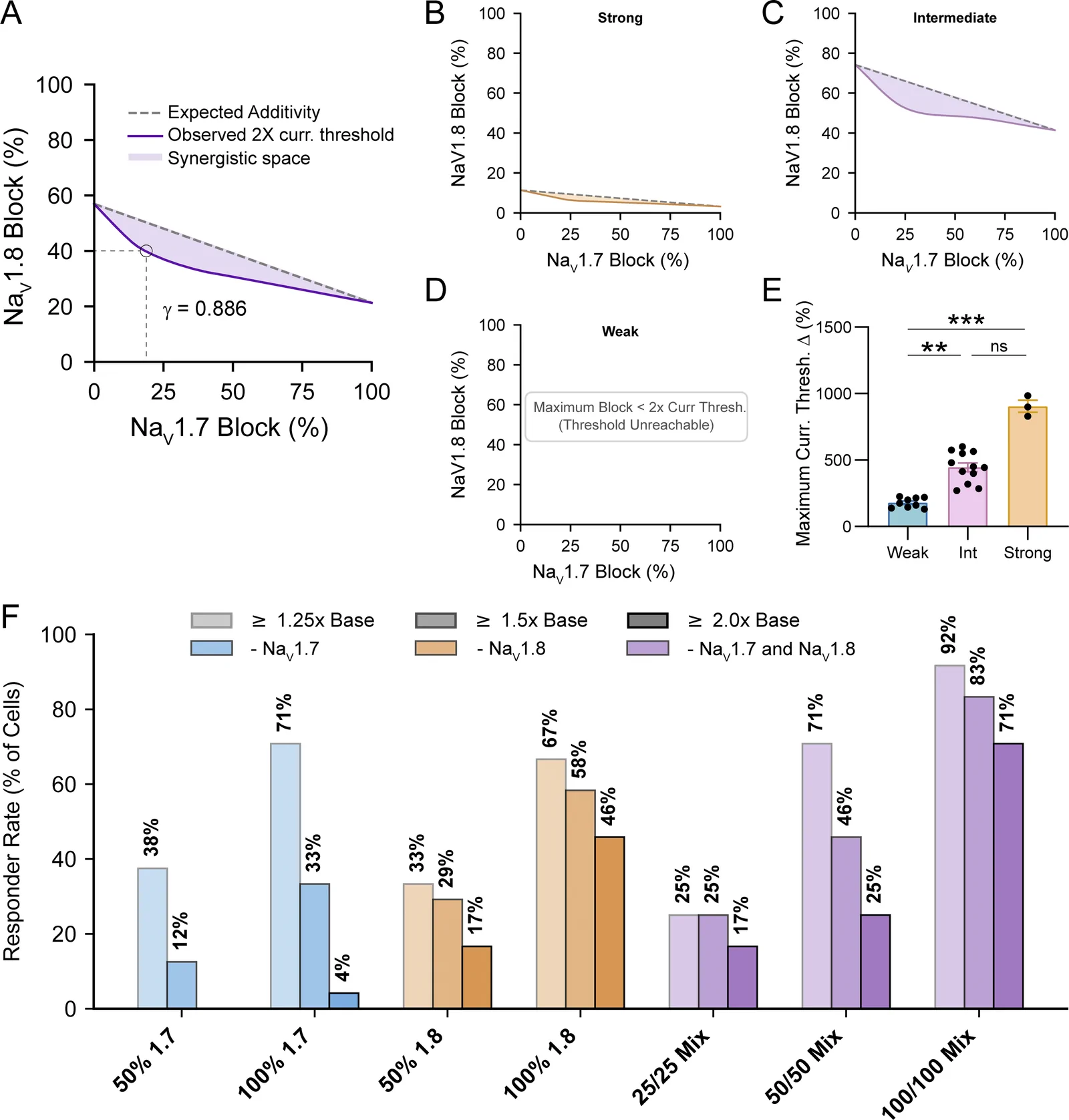
**The potential of Nav1.7/Nav1.8 synergy for dose-sparing analgesia. (A)** Population-level isobologram showing the percent reduction in Nav1.7 and Nav1.8 required to achieve a 2× increase in current threshold. The inward bow of the purple “Observed” curve relative to the “Expected” dashed line highlights the synergistic space. **(B–D)** Isobolograms for neurons within the strong (B), intermediate (C), and weak (D) Nav1.8 response modes. Strong and intermediate responders exhibit inward deviation from the additive expectation (and thus potential dose-sparing), whereas a two-fold current threshold increase was not achieved within the sampled subtraction range in weak responders. **(E)** Maximum current threshold change achieved across the Nav1.7 × Nav1.8 functional subtraction matrix in weak, intermediate, and strong Nav1.8 responders. Bars show mean ± SEM and points represent individual neurons. **(F)** Percentage of neurons achieving current threshold increases of at least 1.25-fold, 1.5-fold, or 2-fold above baseline under 50% or 100% Nav1.7 subtraction alone, 50% or 100% Nav1.8 subtraction alone, or combined 25%/25%, 50%/50%, or 100%/100% Nav1.7/Nav1.8 functional subtraction. Blue bars represent Nav1.7 subtraction alone, orange bars represent Nav1.8 subtraction alone, and purple bars represent combined Nav1.7/Nav1.8 subtraction. Within each color family, progressively darker shades indicate current thresholds of ≥1.25-fold, ≥1.5-fold, and ≥2-fold above baseline. Percentages above each bar indicate the fraction of recorded neurons reaching the corresponding current threshold. **P < 0.01, ***P < 0.001.

To assess the translational implications of variation across the Nav1.8 sensitivity spectrum, we next asked how much Nav1.7 and Nav1.8 functional subtraction was required to produce a defined increase in firing threshold. We therefore constructed isobolograms for a twofold increase in current threshold, using this as a stringent benchmark for strong suppression of excitability. At the population level, the observed isobole bowed markedly inward relative to the independent additive expectation, indicating substantial dose-sparing synergy between Nav1.7 and Nav1.8 attenuation (γ = 0.886; Fig. 8 A). Thus, the same current threshold endpoint could often be reached with considerably less subtraction of each individual conductance when both were reduced together.

However, the extent of this dose-sparing effect varied across the Nav1.8 sensitivity spectrum. Strong and intermediate Nav1.8 responders exhibited pronounced inward-bowed isoboles, consistent with substantial combinatorial vulnerability and efficient dose-sparing (Fig. 8, B and C). In contrast, weak Nav1.8 responders were comparatively resistant, and in many neurons, the twofold current threshold was not reached even with maximal reductions of Nav1.7, Nav1.8, or both conductances (Fig. 8 D). Thus, while combined targeting substantially increased efficacy in neurons that are more responsive to Nav1.8 subtraction, it did not produce equivalent suppression across the full population.

This limit was further evident when the maximal achievable current threshold shift was compared across the Nav1.8 response spectrum (Fig. 8 E). Strong and intermediate responders exhibited substantially larger maximal current threshold shifts than weak responders (weak responders: 178.6 ± 12.4%; intermediate responders: 445.7 ± 32.9%; strong responders: 904.4 ± 45.1%; P < 0.0001, Kruskal–Wallis test). Consistent with this, the fraction of cells reaching progressively larger increases in current threshold depended on the degree and ratio of Nav1.7 and Nav1.8 subtraction (Fig. 8 F).

Together, these results show that dual Nav1.7–Nav1.8 attenuation creates a substantial dose-sparing space, but that the magnitude of this benefit varies across the spectrum of Nav1.8 dependence. In neurons with strong Nav1.8 sensitivity, partial simultaneous attenuation of Nav1.7 and Nav1.8 can produce marked suppression of excitability. In weak Nav1.8 responders, even extensive dual functional subtraction may produce substantially smaller effects, revealing an intrinsic biophysical limit to a therapeutic strategy directed exclusively at these two conductances.

## Discussion

Beginning with the question “if block of Nav1.8 relieves pain only partially, what is needed for better pain relief?,” we have carried out a dynamic clamp study that suggests that combined Nav1.7–Nav1.8 targeting should be expected to outperform monotherapy in attenuating the excitability of some DRG neurons but not to produce universal suppression.

Our results show that peripheral sensory neuron excitability is governed by nonlinear combinatorial interactions within the Nav1.7–Nav1.8 dyad. Clinical studies to date have targeted Nav1.7 and Nav1.8 individually in attempts to provide non-opioid pain relief. Combined targeting of multiple Nav channels has been suggested as a potentially more efficacious approach (Waxman and Vasylyev, 2025). The results of this study indicate that the effects of functional suppression of Nav1.7 and of functional suppression of Nav1.8 on DRG neurons are not independent. Partial simultaneous attenuation of Nav1.7 and Nav1.8 produced a supralinear increase in current threshold in a substantial fraction of neurons, indicating that in these cells the effect of reducing one conductance depends on the remaining availability of the other.

This work extends prior studies that defined complementary roles of peripheral sodium channels Nav1.7 and Nav1.8 in DRG neurons. Nav1.7 contributes prominently near resting and perithreshold voltages, whereas Nav1.8, while contributing at perithreshold voltages in a population of neurons (Vasylyev et al., 2025), generally supports the suprathreshold regenerative and repetitive phases of firing (Vasylyev et al., 2024; Ghovanloo and Waxman, 2026). More recent work showed that Nav1.8-directed perturbation alone encounters an intrinsic ceiling because some neurons are weak responders (Stewart et al., 2025; Vasylyev et al., 2025; Waxman and Vasylyev, 2025). More broadly, prior work has established that the functional effect of an individual conductance depends on the broader complement of conductances within a neuron. Marder and colleagues demonstrated that similar neuronal outputs can arise from substantially different underlying conductance configurations, providing a framework for functional compensation and cell-to-cell variability (Prinz et al., 2004b; Goaillard et al., 2009). Prescott and colleagues further showed that interactions between membrane conductances can be strongly nonlinear, such that the effect of altering one conductance depends on the state of another (Prescott et al., 2006). Our mapping of the Nav1.7–Nav1.8 dyad reveals a spectrum of nonlinear interaction regimes that are similarly context-dependent and nonadditive.

An important finding is that Nav1.7–Nav1.8 functional synergy is not simply present or absent across the population but varies across a spectrum of responses that is strongly related to neuronal sensitivity to Nav1.8 subtraction. Gaussian mixture modeling of responses to complete Nav1.8 subtraction identified weak, intermediate, and strong response modes along this spectrum. We use these modes as a descriptive framework rather than as evidence for discrete sensory neuron subtypes. Neurons with greater sensitivity to Nav1.8 subtraction generally showed greater suppression of excitability during combined Nav1.7–Nav1.8 attenuation, whereas weak Nav1.8 responders remained comparatively resistant. Thus, the efficacy of combined sodium channel suppression appears to depend substantially on the degree to which AP electrogenesis in an individual neuron relies on Nav1.8.

The population current trajectories provide a mechanistic correlate of the Nav1.8 response spectrum identified from the perturbation matrix. Weak responders reached AP threshold while Nav1.8 contributed only a relatively small fraction of the combined Nav1.7 + Nav1.8 sodium current, whereas intermediate and strong responders generally reached threshold at more depolarized voltages where Nav1.8 made a substantially larger contribution. This extends our previous observation that the relative contribution of Nav1.8 in the perithreshold region is linked to AP voltage threshold (Vasylyev et al., 2025) and further indicates that sensitivity to Nav1.8 functional subtraction reflects the voltage-dependent recruitment of Nav1.8 within the broader electrogenic context rather than channel abundance alone. A parsimonious mechanism for the observed synergy is that available Nav1.7 current at relatively hyperpolarized and perithreshold voltages provides depolarization that promotes subsequent recruitment of Nav1.8. Under this model, Nav1.7 attenuation both removes its direct inward current and reduces downstream recruitment of Nav1.8, producing a disproportionately large effect in neurons in which Nav1.8 contributes strongly near threshold. This mechanism is likely simplified, however, because passive membrane properties and other ionic conductances also shape the electrogenic context in which these channels operate. The clear separation of weak responders from the intermediate and strong response modes at threshold therefore provides a biophysical basis for their limited sensitivity to Nav1.8 subtraction and for the greater effect of combined Nav1.7–Nav1.8 attenuation in neurons with stronger functional dependence on Nav1.8.

Our observation that DRG neurons span a broad spectrum of electrogenic responses may also help frame pain heterogeneity (Alsaloum et al., 2025a; Alsaloum et al., 2025b). Transcriptomic and proteomic studies have established that peripheral sensory ganglia contain multiple molecularly distinct neuronal classes (Zheng et al., 2019; Tavares-Ferreira et al., 2022; Barry et al., 2023; Bhuiyan et al., 2024; Bhuiyan et al., 2025, *Preprint*). Although a correlation has not been experimentally demonstrated, it is possible that studies linking molecular identity to electrophysiology might provide a framework for interpreting our findings. In a study on genetically defined mouse DRG classes, Zheng et al. observed subtype-specific AP waveform and firing patterns that tended to correlate with transcriptomic differences and reported greater Nav1.8 expression in small-diameter classes (Zheng et al., 2019). More recently, Patch-seq profiling of human DRG neurons identified three electrophysiological types and mapped them onto specific transcriptional classes (Yi et al., 2025, *Preprint*). Körner et al. similarly combined Patch-seq with transcriptomic analyses to link distinct electrophysiological properties of sensory neurons to molecularly defined classes, identifying a population with features corresponding to human mechano-insensitive (“sleeping”) nociceptors (Körner et al., 2026). Further studies of this type might provide a framework in which the differences in Nav1.8 current amplitude, AP width, and threshold across our response modes might be linked to underlying sensory-neuron identity. It is interesting, in this regard, that microneurography, which permits recording from peripheral sensory axons in human subjects, does not provide direct information about the membrane properties or transcriptomic identity of recorded neurons, but has identified multiple distinct classes of C fibers based on their functional response properties and activity-dependent slowing (Serra et al., 1999; Serra et al., 2004). Our data add an electrogenic layer to this heterogeneity by showing that sensory neurons also differ substantially in their dependence on Nav1.8 and in their response to combined Nav1.7–Nav1.8 attenuation. We do not assign specific pain qualities to different positions along this response spectrum here. However, the results raise the possibility that different pain phenotypes, such as allodynia, punctate pain, or heat pain, may recruit neuronal populations with different electrophysiological vulnerabilities.

There are two translational implications of this work. First, our results show that combined attenuation of Nav1.7 and Nav1.8 creates a substantial dose-sparing space, suggesting that dual targeting of both channels may suppress excitability more effectively than selective inhibition of either isoform alone. This suggests a combinatorial approach that can partially address the incomplete efficacy of selective Nav1.8 inhibition that has been seen in both clinical studies and in recordings from human DRG neurons (Jones et al., 2023; Stewart et al., 2025). Traditional small molecules or emerging modalities such as gene therapy or targeted protein degradation (Tyagi et al., 2025, *Preprint*) could be combined to achieve this dose-sparing space. Our results, however, indicate an important caveat: even dual targeting of Nav1.7 and Nav1.8 was not sufficient to strongly suppress excitability in all neurons. Weak Nav1.8 responders exhibited a more limited maximal current threshold shift, revealing a potential intrinsic biophysical limit to strategies directed exclusively at these two conductances. The important conclusion is that combined Nav1.7–Nav1.8 targeting should be expected to outperform monotherapy in many neurons but not to produce universal suppression. Importantly, universal suppression may be neither necessary nor desirable because DRGs contain non-nociceptive sensory populations. Selective suppression of Nav1.8-sensitive neurons may instead be sufficient for analgesia while preserving other sensory functions.

These findings may also have more general implications for therapeutic strategy. Recent analgesic development has increasingly emphasized precision targeting of one, or at most two, sodium channel isoforms. Our results suggest that even highly selective dual Nav1.7–Nav1.8 targeting may leave some neurons insufficiently suppressed. One likely explanation for weak responsiveness is that AP generation in these neurons is buffered by additional conductances beyond the Nav1.7–Nav1.8 dyad (Mis et al., 2019; Yuan et al., 2021). In that context, safe and tolerable compounds that dampen excitability through actions at a larger universe of channels and receptors may, in some settings, offer a more effective route toward broader pain control (Waxman and Zamponi, 2014; Gordon et al., 2025). Nonpsychotomimetic cannabinoids are one example, since they can modulate excitability through multiple Nav channels as well as additional molecular targets (Zhang et al., 2022; Huang et al., 2023; Ghovanloo et al., 2025). As a second example, lacosamide, which is functionally selective for inactivated channels but not subtype-specific, has been shown to ameliorate pain in peripheral neuropathy (Greef et al., 2019). This does not argue against precision target-specific pharmacology but suggests that for diverse pain states, which may recruit sensory neurons with different electrogenic dependencies, rationally designed or carefully selected polypharmacology may prove advantageous.

This study has several limitations. Firstly, the number of neurons studied (*n* = 24) was constrained by the technical demands of the dynamic clamp protocol, which required 20 current threshold measurements and at least 40–50 min of stable recording from each neuron. This sample size is not sufficient to establish discrete electrophysiological subtypes, and we therefore interpret the weak, intermediate, and strong Nav1.8 response modes as a descriptive framework for the spectrum of responses observed here. Larger datasets will be required to determine whether these modes represent stable biological populations. In addition, recordings were obtained from small-diameter male P4–P5 rat DRG neurons. These neurons exhibited endogenous Nav1.7 and Nav1.8 currents and are well suited for examining the functional interaction between these channels. Because only male animals were studied, sex could not be examined as a biological variable. Developmental and species differences in ion channel expression and excitability may also limit extrapolation of these findings to adult or human sensory neurons, and the extent to which the response spectrum and Nav1.7–Nav1.8 interactions described here are conserved in these populations remains to be determined. Finally, the perturbation matrix necessarily interrogated only two conductances within a broader excitability network. The voltage-based separation provided an operational estimate of the Nav1.8-dominant TTX-resistant current rather than pharmacological confirmation of channel identity. Incorporating subtype-selective inhibitors into the already prolonged recording protocol would have increased the potential for recording drift, channel rundown, cumulative drug exposure, and incomplete washout. Moreover, Nav1.7 was estimated as 70% of the TTX-sensitive current, and the molecular contributors to the remaining TTX-sensitive component were not determined. Additional sodium, potassium, calcium, and other membrane conductances are likely to contribute to the relative resistance observed in weak Nav1.8 responders. In particular, Nav1.9 expression and functional contribution were not directly assessed. Because Nav1.9 produces a persistent, low-threshold inward current, it could help maintain excitability following Nav1.7/Nav1.8 subtraction, particularly in weak Nav1.8 responders. A logical next step will be to determine which additional conductances support excitability in weak Nav1.8 responders. Despite these limitations, however, the present results directly reveal nonlinear interactions between Nav1.7 and Nav1.8 that are inaccessible to studies of single-isoform perturbations alone and demonstrate substantial cell-to-cell variability in the functional consequences of their attenuation.

In summary, our results show that combinatorial functional abrogation of Nav1.7 and Nav1.8 affects peripheral sensory neuron excitability in a nonlinear manner. Partial dual attenuation of both Nav1.7 and Nav1.8 can produce profound supralinear suppression in some neurons, yet even strong abrogation of both Nav1.7 and Nav1.8 currents remains insufficient in others. Responses to Nav1.8 functional subtraction span a broad spectrum, with Nav1.8 dependence emerging as a major determinant of neuronal sensitivity to combined channel attenuation, while Nav1.7 provides a comparatively uniform and functionally cooperative contribution. These findings provide a biophysical framework for understanding both the potential dose-sparing benefit and the limitations of combined Nav1.7–Nav1.8 targeting.

## Data Availability

All data in this study are available from the corresponding author upon reasonable request.
